# Traditional Chinese exercises on muscle strength, muscle mass, and physical function in older adults with sarcopenia: a systematic review and meta-analysis

**DOI:** 10.3389/fmed.2026.1841875

**Published:** 2026-08-05

**Authors:** Ruidie Ma, Lei Gou, Si Ma, Yingbing Mei, Xueliang Wang, Wang Zhu, Yu Guo

**Affiliations:** 1 Hubei University of Chinese Medicine, Wuhan, Hubei, China; 2 Yuntianhua Group Co., Ltd. Shuifu Branch, Zhaotong, Yunnan, China; 3 Hubei Provincial Hospital of Traditional Chinese Medicine, Wuhan, Hubei, China; 4 Zhaotong Hospital of Traditional Chinese Medicine, Zhaotong, Yunnan, China

**Keywords:** meta-analysis, muscle mass, muscle strength, older adults, physical function, sarcopenia, traditional Chinese exercises

## Abstract

**Objective:**

This study aimed to systematically evaluate the effects of Traditional Chinese Exercises (TCEs) on muscle strength, muscle mass, and physical function in older adults with sarcopenia.

**Methods:**

Systematic electronic searches were conducted in PubMed, Embase, Web of Science, Cochrane Library, CBM, CNKI, VIP and Wanfang Database from inception to March 1, 2026, to identify randomized controlled trials (RCTs) assessing the efficacy of TCEs in older adults with sarcopenia. Meta-analyses were performed using RevMan 5.4, and effect sizes were expressed as mean difference (MD) with 95% confidence interval (CI).

**Results:**

Seventeen studies involving 984 participants were included. The meta-analysis revealed that TCEs were associated with improvements in handgrip strength (HS) (MD = 1.13, 95% CI: 0.94–1.32, *p* < 0.001, *I*^2^ = 2%). Regarding muscle mass, TCEs were also associated with increases in appendicular skeletal muscle mass index (ASMI) (MD = 0.20, 95% CI: 0.11–0.29, *p* < 0.001, *I*^2^ = 0%). For physical function, TCEs were associated with improvements in 6-meter gait speed (6m_GS) (MD = 0.12, 95% CI: 0.09–0.16, *p* < 0.001, *I*^2^ = 0%), timed up-and-go test (TUGT) performance (MD = −2.80, 95% CI:−2.55–−2.05, *p* < 0.001, *I*^2^ = 0%), five-times sit-to-stand test (FTSST) (MD = −1.37, 95% CI:−1.89–−0.85, *p* < 0.001, *I*^2^ = 0%), 30-s sit-to-stand test (30s STS) (MD = 1.53, 95% CI: 0.64–2.24, *p* < 0.001, *I*^2^ = 0%), and short physical performance battery (SPPB) (MD = 1.37, 95% CI: 0.81–1.92, *p* < 0.001, *I*^2^ = 0%). Subgroup analyses based on exercise modality, intervention duration, frequency, and session length showed no statistically significant differences across outcomes among different subgroups (*p* > 0.05).

**Conclusion:**

TCEs may offer potential benefits for improving muscle strength, mass, and physical function in older adults with sarcopenia. However, these findings should be interpreted with caution due to the limited number of studies, methodological limitations (e.g., lack of blinding), small sample sizes, and clinical heterogeneity across the included trials. High-quality, large-scale RCTs are warranted to confirm these findings and strengthen the evidence base.

**Systematic review registration:**

https://www.crd.york.ac.uk/PROSPERO/view/CRD420261343216, identifier: CRD420261343216.

## 1 Introduction

Sarcopenia is an age-related syndrome that is highly prevalent among older adults and is characterized by progressive declines in skeletal muscle mass, strength, and physical function (1, 2). Epidemiological data indicate that its prevalence increases with age, ranging from 5% to 13% in individuals aged 60–70 years and from 11% to 50% in those aged ≥80 years (3, 4). In 2010, the European Working Group on Sarcopenia in Older People (EWGSOP) established diagnostic criteria encompassing muscle mass, muscle strength, and physical function. Specifically, low muscle mass (LMM) is defined as a skeletal muscle mass index (SMI) of less than 8.90 kg/m^2^; low muscle strength (LMS) is defined as handgrip strength of less than 30 kg for men and 20 kg for women; and low physical performance (LPP) is defined as gait speed of less than 0.8 m/s. The diagnosis of sarcopenia requires the presence of low muscle mass in combination with either low muscle strength or low physical function (5). Clinically, sarcopenia commonly presents with slowed gait and difficulty in standing. It also significantly increases the risk of falls and related fractures, as well as the likelihood of disability, hospitalization, and even mortality in older adults (6, 7). Evidence suggests that sarcopenia may indirectly exacerbate the financial and healthcare burden on families and society by impairing patients' ability to perform activities of daily living (6). Therefore, early prevention and timely intervention for sarcopenia are of critical importance in both clinical practice and public health.

Exercise is widely recognized as an effective non-pharmacological strategy for sarcopenia, although no specific pharmacological treatments are currently approved for its management (8–11). Contemporary rehabilitation strategies, including resistance, aerobic, and balance training, have constituted the mainstay of exercise interventions for sarcopenia in older adults. Among these modalities, resistance training is the most commonly recommended, as it promotes muscle protein synthesis and enhances muscle mass and strength (12). However, older adults may have difficulty maintaining long-term adherence due to reduced physical capacity, joint discomfort, or poor exercise compliance, and such training often requires specialized equipment, facilities, and professional supervision. Indeed, studies have shown that adherence among older adults declines markedly in the latter half of interventions, even in technology-supported home-based resistance training programs, adherence remains suboptimal (13). Accordingly, Traditional Chinese Exercises (TCEs) have gained increasing popularity among older adults in recent years owing to their moderate intensity, low cost, low risk, and integration of physical and mental regulation.

TCEs originated from long-term traditional Chinese health and physical practices, such as Tai Chi, Baduanjin, Yi Jin Jing, and others. They take several forms, including routines and qigong exercises, and are focused on maintaining health. Traditional philosophical ideas like yin-yang, the five elements, and essence-qi-spirit have had a significant impact on their development. As a result of widespread medical practice, they have progressively transformed into therapeutic exercise methods with rehabilitative purposes (14, 15). TCEs are a fundamental intervention for sarcopenia in older adults in clinical practice, although there is conflicting evidence about the best kind of exercise. Although an increasing number of systematic reviews and meta-analyses have studied the health benefits of TCEs for sarcopenia in older adults, there are still certain limitations. Existing evidence suggests that TCEs may improve muscle strength and physical function in older adults with sarcopenia (16). However, the effects on muscle mass have not been consistently evaluated across different types of TCEs. This limitation hinders the development of evidence-based exercise prescriptions and leaves uncertainty regarding the optimal type of exercise for improving muscle mass. In addition, the relatively small sample sizes of included primary studies may reduce the robustness and generalizability of the available evidence. Moreover, one previous study relied on a pre-post comparison within the intervention group rather than a direct comparison between intervention and control groups (17), which may reduce the certainty of the evidence by limiting causal inference and failing to adequately account for confounding factors, such as temporal impacts and the disease's natural development.

To this end, the present study aims to conduct a meta-analysis based on direct comparisons between intervention and control groups to systematically evaluate the effects of TCEs in older adults with sarcopenia, focusing on key clinical outcomes, including muscle strength, muscle mass, and physical function. This may provide more robust evidence to inform exercise prescription strategies for this population.

## 2 Materials and methods

### 2.1 Registration of systematic review protocol

To guarantee transparency, methodological rigor, and reproducibility of the evidence synthesis, this systematic review and meta-analysis are reported in compliance with the Preferred Reporting Items for Systematic Reviews and Meta-Analyses (PRISMA) 2020 statement (18) (Supplementary File S1). The study protocol was prospectively registered with the International Prospective Register of Systematic Reviews (PROSPERO) (registration number: CRD420261343216) (Supplementary File S2), outlining its goals, eligibility requirements, search strategy, and planned methods of analysis.

### 2.2 Identification and selection of studies

From inception to March 1, 2026, the following electronic databases were searched: PubMed, Cochrane Library, Embase, Web of Science, Chinese Biomedical Literature Database (CBM), CNKI, VIP, and Wanfang Database. Keywords related to the study population (e.g., sarcopenia, older adults) and interventions (e.g., Tai Chi, Baduanjin, Traditional Chinese Exercises) were incorporated into the search strategy. The PubMed search strategy was adapted for use across other databases. To identify additional eligible studies, the reference lists of all included articles were hand-searched. Potentially relevant studies were retrieved in full text for further assessment. Titles and abstracts were independently screened for eligibility. Final inclusion decisions were resolved through consensus discussions among all reviewers. Detailed search strategies are provided in the Supplementary File S3.

### 2.3 Eligibility criteria

The eligibility criteria were as follows: (1) Study population: Study population: older adults with sarcopenia of any gender, with a study-level mean age ≥60 years (as defined by accepted diagnostic criteria or guidelines); (2) Intervention: TCEs, including Tai Chi, Baduanjin, and Yi Jin Jing, were applied in the intervention group; (3) Control group: interventions such as health education or usual care were used in the control group; (4) Outcomes: studies were required to report at least one of the following outcomes, including handgrip strength (HS, as a measure of muscle strength), appendicular skeletal muscle mass index (ASMI, as a measure of muscle mass), or physical function outcomes, including the five-times sit-to-stand test (FTSST), 30-s sit-to-stand test (30s STS), 6-meter gait speed (6m_GS), timed up-and-go test (TUGT), or Short Physical Performance Battery (SPPB); (5) Study type: randomized controlled trials (RCTs) only.

The exclusion criteria were as follows: (1) Publication type: non-original research articles, including reviews, case reports, systematic reviews, and meta-analyses; (2) Combined interventions: studies combining TCEs with other exercise modalities (e.g., cycling, swimming, or interval training), or studies with unclear intervention classification; (3) Insufficient intervention duration: exclusion of acute or single-session intervention studies; (4) Incomplete or inaccurate data: studies with irretrievable or incomplete data despite attempts to contact authors, or those with obvious methodological flaws or inconsistencies; (5) Duplicate or overlapping publications: duplicate publications or studies using overlapping datasets without additional novel findings.

### 2.4 Data extraction

The following data were independently extracted by two authors (RM and LG): (1) Study characteristics (year of publication and geographical location) and participant characteristics (sample size, sex, and age); (2) intervention and control conditions; (3) primary outcomes; and (4) effect estimates for the outcomes of interest. For quantitative synthesis, sample sizes and outcome data, including means and standard deviations (SDs), or mean difference (MDs) with 95% confidence intervals (CIs), were extracted for both intervention and control groups. All data were entered in a predesigned Microsoft Excel spreadsheet (Microsoft Corporation, Redmond, WA, USA). Data extraction was independently verified by the authors, and any discrepancies were resolved through discussion and consensus. When SDs were not reported, they were calculated from standard errors, CIs, t values, or *p* values. For missing or incomplete data, at least three attempts were made to contact the corresponding authors via email.

### 2.5 Risk of bias assessment

Two reviewers (RM and LG) independently assessed the risk of bias in the included studies using the Cochrane Risk of Bias 2.0 (RoB 2) tool (19, 20). Any discrepancies were resolved through discussion, and when necessary, a third reviewer was consulted to reach a consensus. This procedure ensured consistency in the risk of bias assessment of the included RCTs. The risk of bias was evaluated across the following domains: random sequence generation (selection bias), allocation concealment (selection bias), blinding of participants and personnel (performance bias), blinding of outcome assessment (detection bias), incomplete outcome data (attrition bias), selective reporting (reporting bias), and other potential sources of bias. Each domain was classified as low risk, some concerns, or high risk.

### 2.6 Assessment of certainty of evidence

The GRADE (Grading of Recommendations Assessment, Development and Evaluation) method was used to assess the degree of certainty of the evidence for each outcome, taking into account publication bias, risk of bias, inconsistency, indirectness, and imprecision (21). The evaluation was completed independently by two reviewers (RM and LG), and any disagreements were settled through conversation.

### 2.7 Statistical analysis

Using RevMan 5.4 and Stata 16 software, meta-analyses were performed to evaluate the effects of TCEs on sarcopenia in older adults and to explore intervention characteristics. All outcomes in the included trials were continuous variables. The pooled MD with 95% CI was used as the effect size when measurement units were consistent; otherwise, standardized MD with 95% CI were applied. Statistical heterogeneity was assessed using the *I*^2^ statistic and the chi-square (Q) test. A fixed-effects model was used as the primary analytical approach when statistical heterogeneity was low (*I*^2^ ≤ 50% and *P* ≥ 0.10). However, given the potential clinical heterogeneity inherent in exercise-based interventions, including variations in exercise modality, intervention duration, frequency, and participant characteristics, random-effects models were additionally performed in sensitivity analyses to assess the robustness of the findings. Subgroup analyses were conducted to explore the potential influence of exercise method, intervention duration, intervention frequency, and session duration on outcome measures. All analyses were two-tailed, and a *P* value <0.05 was considered statistically significant.

## 3 Results

### 3.1 Study selection

The database searches identified a total of 474 records, including PubMed (*n* = 45), Web of Science (*n* = 73), Embase (*n* = 56), Cochrane Library (*n* = 180), CNKI (*n* = 24), Wanfang Database (*n* = 50), VIP (*n* = 23), and CBM (*n* = 23). An additional 9 records were identified through other sources. After removing duplicates (*n* = 124), 350 records remained for screening.

**Figure 1:**
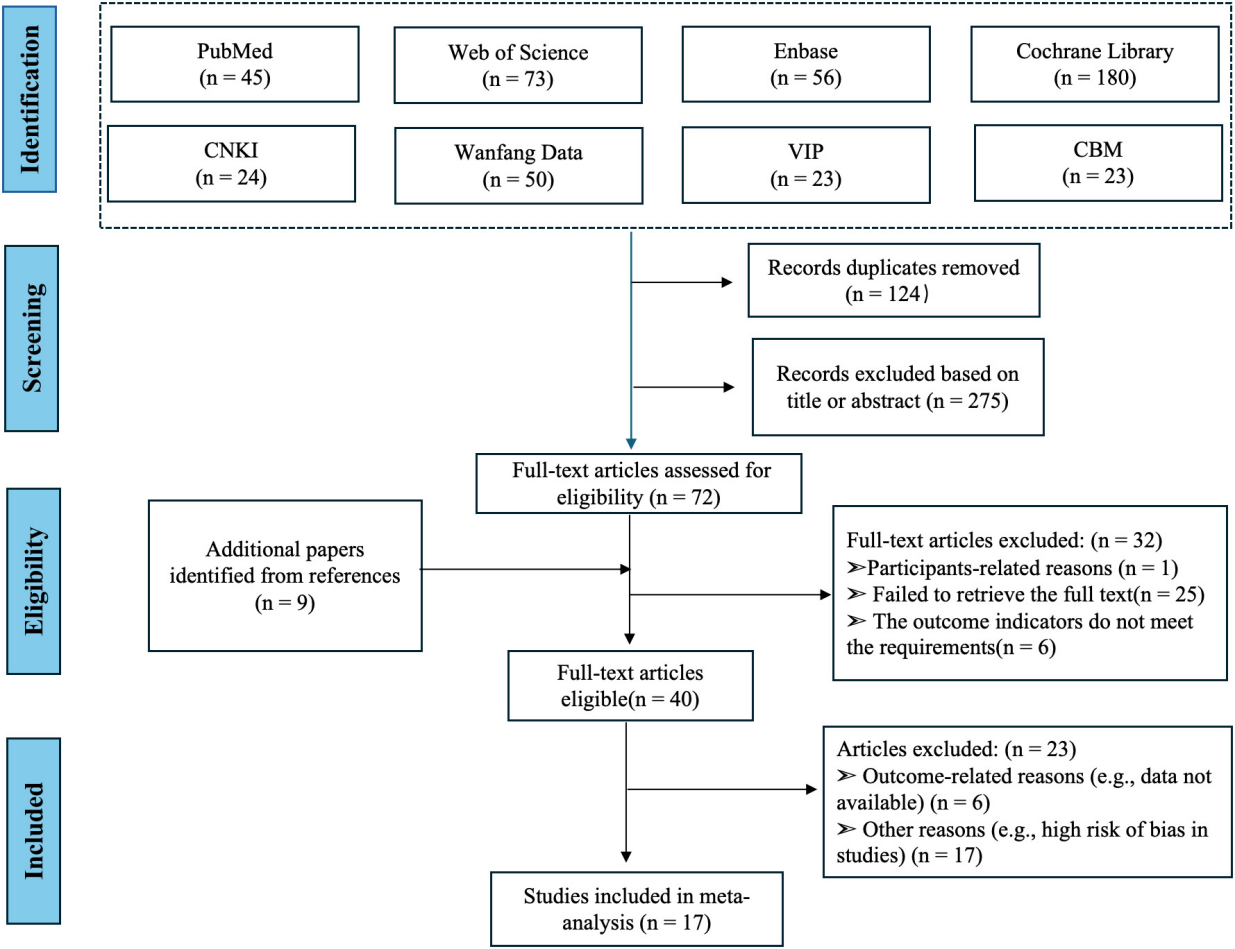
PRISMA flow diagram of the study selection.

Following title and abstract screening, 72 records were assessed for full-text eligibility. Of these, 32 full-text articles were excluded for the following reasons: lack of eligible outcome measures (*n* = 6), full texts unavailable (*n* = 25), and ineligible population characteristics (*n* = 1). For the 25 studies with unavailable full texts, multiple attempts were made to obtain the articles, including contacting journal publishers, corresponding authors, and searching institutional databases. However, the full texts could not be retrieved, and these studies were excluded. Ultimately, 17 studies were included in the meta-analysis (Figure 1, Supplementary File S4, Supplementary Figure S1).

### 3.2 . Methodological quality

**Figure 2:**
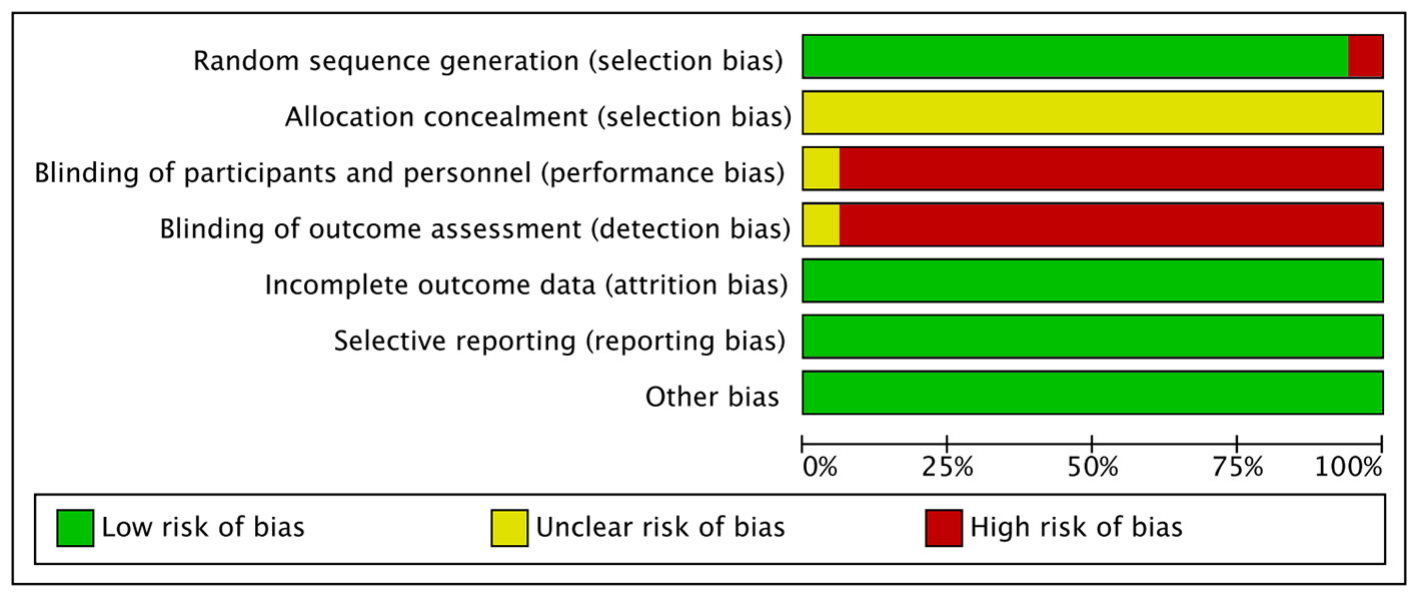
RoB2 risk of bias ratings for each eligible study.

The results of the risk of bias assessment of the included studies are presented in (Figure 2, Supplementary Figure S2). Bias was assessed across five domains relevant to RCTs. Overall, 80–90% of the included studies were judged to have a low risk of bias in random sequence generation and allocation concealment. These findings indicated that most of the included trials employed appropriate randomization methods, thereby ensuring adequate methodological rigor and comparability between intervention groups.

However, on the dimensions of the “blinding of participants and personnel” and “blinding of outcome assessment,” only approximately 10% of the studies were rated as having an unclear risk of bias, suggesting that most studies exhibited methodological limitations or omissions in blinding procedures, which may have affected the objectivity of the study findings. In contrast, all included studies were judged to have a low risk of bias in incomplete outcome data.

Overall, the methodological quality of the included studies was generally acceptable; however, some uncertainty remained regarding the implementation of blinding. Therefore, caution should be exercised when interpreting the findings, particularly in relation to potential bias introduced by study design limitations.

**Figure 3:**
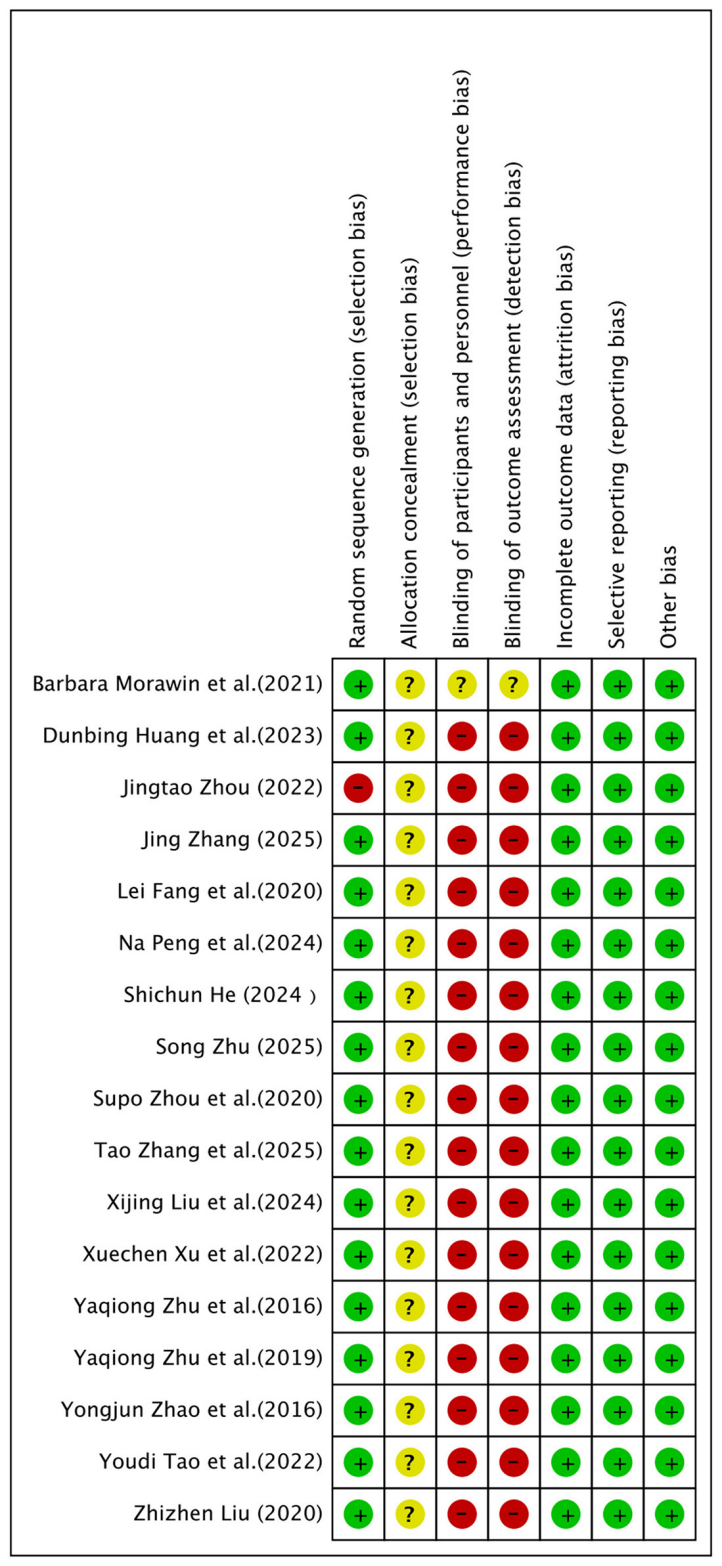
The assessment of each risk of bias item in each study included in the risk of bias summary.

The methodological quality of the 17 included RCTs was assessed using the RoB 2 tool (Figure 3, Supplementary Figure S3). For random sequence generation, 16 studies clearly reported the use of appropriate randomization methods (e.g., computer-generated randomization or random number tables), whereas one study did not provide sufficient details regarding the allocation process. Given the nature of exercise interventions, blinding of participants and personnel is inherently challenging. Among the included studies, all but one did not clearly report whether blinding was implemented, and most studies did not apply blinding procedures, which may have introduced a risk of performance bias.

### 3.3 . Study characteristics

A total of 984 participants (503 women), with a mean age of 62.15±3.39 years, were included across the 17 studies. Of these, 491 participants were assigned to the intervention group and 493 to the control group. The reported outcome measures included HS, 6m_GS, ASMI, TUGT, FTSST, 30s STS, and SPPB. Study characteristics are summarized in Table 1. Among the included studies, 13 reported HS, 7 reported 6m_GS, 8 reported ASMI, 5 reported TUGT, 3 reported FTSST, 2 reported 30s STS, and 3 reported SPPB. The studies were conducted in China and Poland. The intervention duration of the included trials ranged from 4 to 72 weeks, with session durations of 25–60 min and frequencies of 3 to 5 sessions per week. The interventions included Tai Chi, Yi Jin Jing, and Baduanjin, while control groups received health education, usual care, or similar interventions.

**Table 1:** Characteristics of the RCTs included for the systematic review.

References Country	Sample size	Mean age in years ±standard deviation	Intervention characteristics	Control	Evaluating indicators	Measurement time points	Flow up
Morawin et al. (22) Poland	E: 40	E:71.3 ± 7.55	Tai Chi	Health education	a, b, c	24w	NR
	C: 40	C:71.9 ± 4.16	Freq: twice a week				
			Duration: 40 min				
			Course: 24 w				
Zhou (23) China	E:13	E:82.22 ± 3.59	Baduanjin	Nonintervention	a, b, c, d, e	12w	NR
	C:13	C: 81.0 ± 4.0	Freq: 3 times a week				
			Duration: 60 min				
			Course: 12w				
Zhang (24) China	E: 18	E:87.38 ± 3.72	Baduanjin	Health education	a, b, c, f	12w	NR
	C: 18	C:85.69 ± 4.60	Freq: 3 times a week				
			Duration: 30 min				
			Course: 12w				
Peng et al. (25) China	E: 63	E:70.65 ± 5.78	Baduanjin	Conventional therapy	a, d	12w	NR
	C: 63	C:70.88 ± 6.02	Freq: 5 times a week				
			Duration: 30 min				
			Course: 12w				
He (26) China	E: 23	E:70.91 ± 3.94	Tai Chi	Remote guidance	a, b, c	12w	NR
	C: 23	C:72.26 ± 4.43	Freq: 3 times a week				
			Duration: 40 min				
			Course: 12w				
Zhu (27) China	E: 16	E:73.56 ± 4.38	Baduanjin	Resistance training	a, c, e	12w	NR
	C: 16	C:73.19 ± 3.65	Freq: 3 times a week				
			Duration: 60 min				
			Course: 12w				
Zhou et al. (28) China	E: 20	E:72.67 ± 9.56	Baduanjin	Non intervention	a, f	8w	NR
	C: 20	C:73.25 ± 8.54	Freq: 5 times a week				
			Duration: 40 min				
			Course: 8w				
Zhang et al. (29) China	E: 60	E: 72.7 ± 4.8	Yi Jin Jing	Non intervention	a, c, f	12w	NR
	C: 60	C: 73.2 ± 4.6	Freq: 5 times a week				
			Duration: 30 min				
			Course: 12w				
Liu et al. (30) China	E: 60	E:62.15 ± 3.39	Baduanjin	Routine care	a	4w	NR
	C: 60	C:62.74 ± 3.06	Freq: 3 times a week				
			Duration: 30 min				
			Course: 4w				
Xu et al. (31) China	E: 30	E:70.63 ± 0.85	Baduanjin	Conventional therapy	a	12w	NR
	C: 30	C:70.73 ± 0.76	Freq: 3 times a week				
			Duration: 30 min				
			Course: 12w				
Zhu et al. (32) China	E: 24	E: 88.8 ± 3.7	Tai Chi	Health education	a	8w	NR
	C: 27	C: 87.5 ± 3.0	Freq: 5 times a week				
			Duration: 40 min				
			Course: 8w				
Zhao et al. (33) China	E: 6	E: 67.8 ± 3.8	Yi Jin Jing	Non intervention	a, b, c	8w	NR
	C: 6	C: 66 ± 3.11	Freq: 3 times a week				
			Duration: 40 min				
			Course: 8w				
Liu (34) China	E: 18	E:68.53 ± 5.26	Tai Chi	Health education	a, b, d, e	12w	NR
	C: 18	C:67.57 ± 5.51	Freq: 3 times a week				
			Duration: 60 min				
			Course: 12w				
Tao et al. (35) China	E: 37	E: 76.0 ± 3.0	Tai Chi	Conventional therapy	b, c	12w	NR
	C: 36	C: 77.0 ± 3.0	Freq: 3 times a week				
			Duration: 60 min				
			Course: 12w				
Fang et al. (56) China	E: 18	E: 82.5 ± 8.5	Yi Jin Jing	Health education	e	24w	NR
	C: 18	C: 76.3 ± 9.9	Freq: 3 times a week				
			Duration: 25–30				
			min Course: 24w				
Huang et al. (36) China	E: 17	E:72.59 ± 4.27	Baduanjin	Nonintervention	e, f	12w	NR
	C: 18	C:71.50 ± 3.37	Freq: 3 times a week				
			Duration: 50 min				
			Course: 12w				
Zhu et al. (37) China	E: 28	E: 64.0 ± 3.0	Tai Chi	Non intervention	f	72w	NR
	C: 27	C: 64.0 ± 4.0	Freq: 5 times a week				
			Duration: 60 min				
			Course: 72w				

E, Experimental group; C, Control group; NR, No record; a, Grip strength; b, 6-meter gait speed; c, Appendicular skeletal muscle mass index; d, Short Physical Performance Battery (SPPB); e, Timed Up and Go test (TUGT); f, Chair stand test.

**Figure 4:**
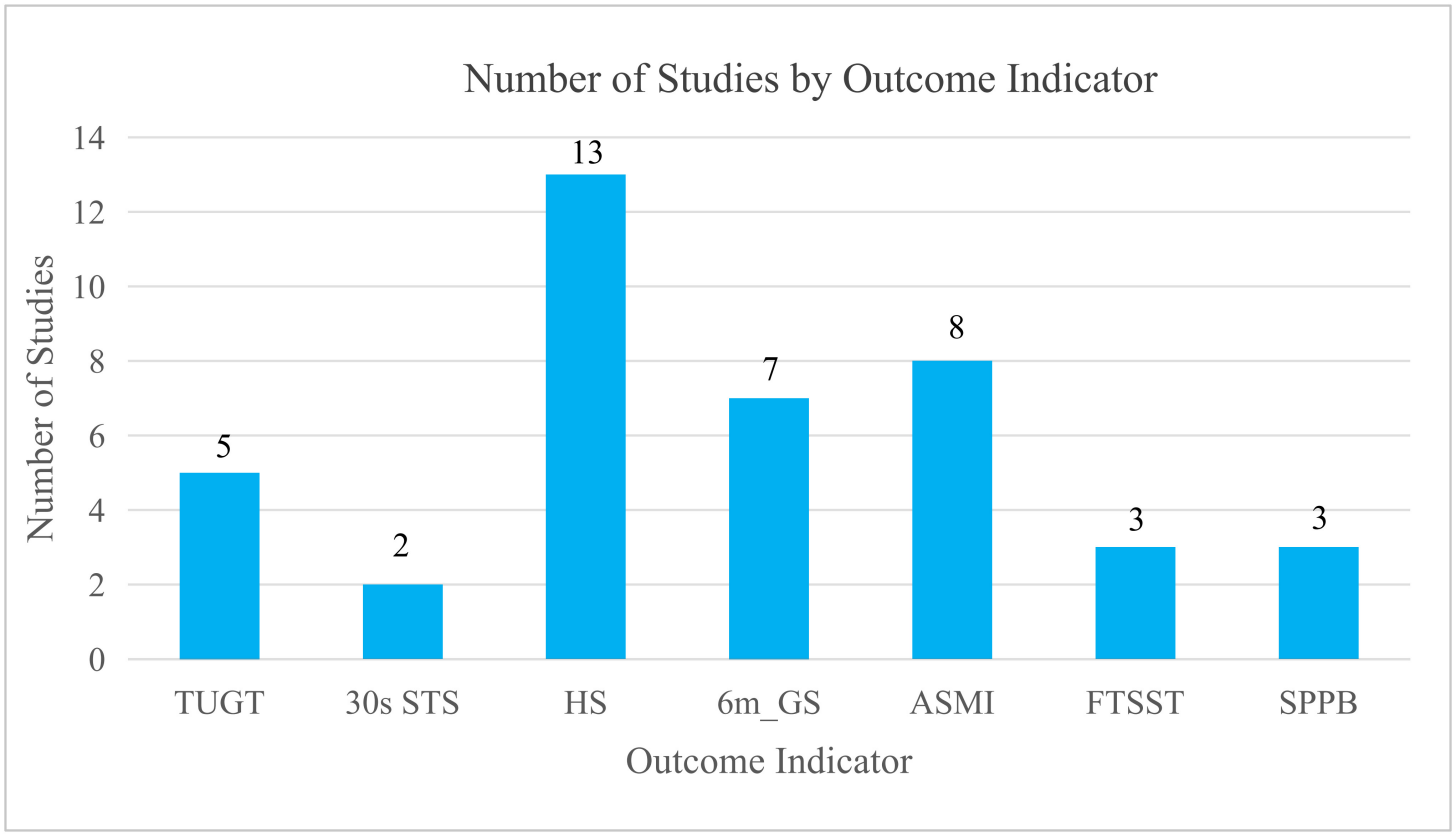
Number of included studies corresponding to each primary outcome indicator. TUGT = timed up and go test, 30s STS = 30-s sit-to-stand test, HS = handgrip strength, 6m_GS = 6-meter gait speed, ASMI = appendicular skeletal muscle mass index, FTSTS = five-times sit-to-stand test, SPPB = short physical performance battery.

A bar chart (Figure 4, Supplementary Figure S4) illustrates the distribution of studies across each primary outcome (HS, ASMI, 6m_GS, FTSST, 30s STS, and SPPB), providing a visual summary of evidence contribution. This facilitates a clearer understanding of the available evidence supporting each outcome and allows rapid assessment of the breadth of data across the included studies. A brief description is provided in the figure legend.

### 3.4 Results of the meta-analysis

#### 3.4.1 Handgrip strength

**Figure 5:**
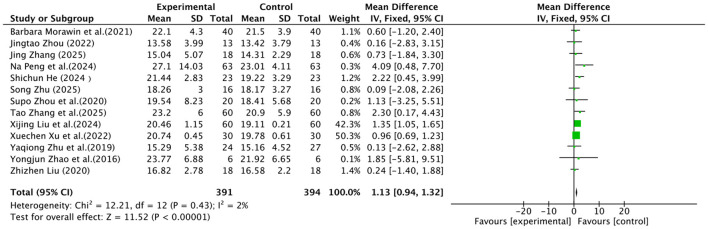
Forest plot of the effects of traditional Chinese exercises on handgrip strength in older adults with sarcopenia.

A total of 13 RCTs, comprising 785 participants (391 in the experimental group and 394 in the control group) (22–34), reported the effects of TCEs on HS in older adults with sarcopenia. The studies' modest heterogeneity (*I*^2^ = 2%, *P* = 0.43) supported the application of a fixed-effects model. TCEs improved HS in this cohort, according to the pooled results (MD = 1.13, 95% CI: 0.94–1.32, *p* < 0.001) (Figure 5, Supplementary Figure S5). Sensitivity analyses suggested that the findings were robust and not materially altered (Supplementary Figures S11, S11.1–S11.4). Egger's test showed that there was no publication bias (*p* = 0.978 > 0.05). Exercise type subgroup analyses showed that Yi Jin Jing (MD = 2.27, 95% CI: 0.22–4.32, *p* = 0.03) and Baduanjin (MD = 1.13, 95% CI: 0.93–1.13, *p* < 0.001) improved HS, while Tai Chi (MD = 0.88, 95% CI:−0.06–1.82, *p* = 0.07) (Table 2). Subgroup analyses based on the frequency and duration of the intervention revealed no significant moderating effects on the results (*p* > 0.05).

**Table 2:** Analysis of the intervention effects of traditional Chinese exercises on various outcome measures in older adults with sarcopenia under different intervention parameters.

Outcomes	Subgroup	K	MD	95%CI	*P*	*I* ^2^
Handgrip strength	Exercise methods				0.48	
	Tai Chi	213	0.88	[−0.06, 1.82]	0.07	5%
	Baduanjin	440	1.13	[0.93, 1.13]	<0.001	21%
	Yi Jin Jing	132	2.27	[0.22, 4.32]	0.03	0%
	Length (weeks)				0.07	
	≤ 8	223	1.34	[1.04, 1.63]	<0.001	0%
	>8	562	0.98	[0.72, 1.23]	<0.001	2%
	Frequency (sessions/week)				0.32	
	≤ 3	448	1.12	[0.92, 1.31]	<0.001	0%
	>3	337	1.86	[0.42, 3.30]	0.01	8%
	Session duration (minutes)				0.33	
	≤ 30	462	1.15	[0.96, 1.35]	<0.001	46%
	>30	223	0.74	[−0.07, 1.55]	0.07	0%
6–Meter walk test	Exercise methods				0.50	
	Tai Chi	235	0.11	[0.07, 0.16]	<0.001	0%
	Baduanjin	62	0.14	[0.08, 0.20]	<0.001	0%
	Session duration (minutes)				0.14	
	≤ 40	174	0.08	[0.02, 0.14]	0.01	0%
	>40	135	0.14	[0.10, 0.18]	<0.001	0%
Appendicular skeletal muscle mass index	Exercise methods				0.50	
	Tai Chi	199	0.25	[0.12, 0.38]	<0.001	0%
	Baduanjin	94	0.14	[0.00, 0.28]	0.05	0%
	Yi Jin Jing	132	0.24	[−0.07, 0.55]	0.13	0%
	Session duration (minutes)				0.63	
	≤ 40	294	0.17	[0.03, 0.32]	0.02	0%
	>40	131	0.22	[0.10, 0.33]	<0.001	0%
Timed up and go test	Session duration (minutes)				0.98	
	≤ 40	76	−2.81	[-4.04, −1.59]	<0.001	0%
	>40	97	−2.79	[−3.75, −1.83]	<0.001	0%
Sit–to–Stand Test	Assessments				<0.001	
	FTSST	126	−1.37	[−1.89, −0.85]	<0.001	0%
	30s STS	160	1.53	[0.64, 2.42]	<0.001	0%

ASMI, TUGT, FTSST, 30s STS, and SPPB, were rated as low to moderate certainty. The main reasons for downgrading were risk of bias, imprecision due to small sample sizes, and limited generalizability, despite low statistical heterogeneity (Supplementary File S5).

#### 3.4.2 6-Meter gait speed

**Figure 6:**
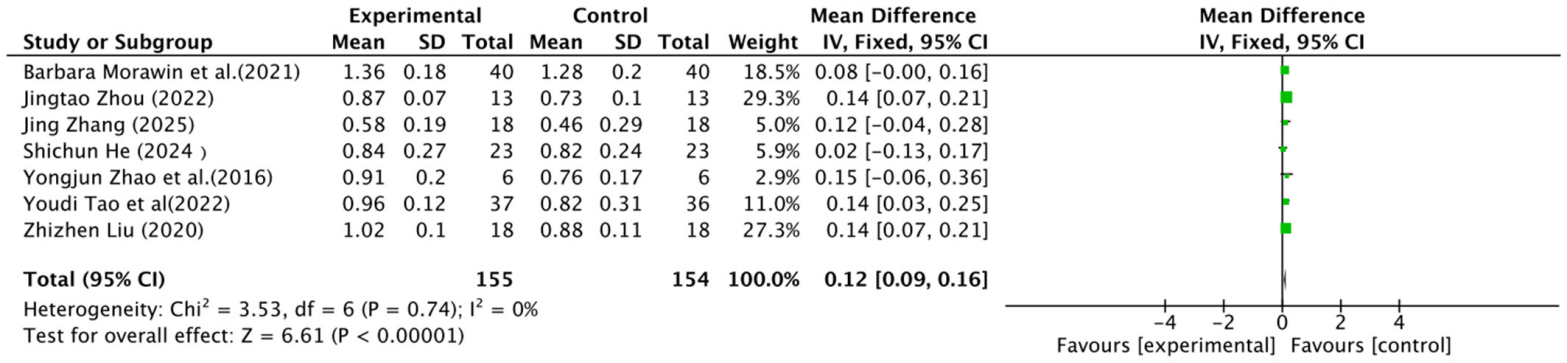
Forest plot of the effects of traditional Chinese exercises on the 6-meter walk test in older adults with sarcopenia.

A total of 7 RCTs, comprising 309 participants (155 in the experimental group and 154 in the control group) (22, 24, 27, 29, 30, 33, 35) reported the effects of TCEs on 6m_GS in older adults with sarcopenia. The studies' modest heterogeneity (*I*^2^ = 0%, *P* = 0.74) supported the application of a fixed-effects model. TCEs improved 6m_GS in this cohort, according to the pooled results (MD = 0.12, 95% CI: 0.09–0.16, *p* < 0.001) (Figure 6, Supplementary Figures S6). Sensitivity analyses suggested that the findings were robust and not materially altered (Supplementary Figures S12, S12.1-S12.3). Egger's test showed that there was no publication bias (*p* = 0.542 > 0.05). There was just one study on Yi Jin Jing intervention because of the small amount of literature that was included in the evaluation of this outcome indicator. Additionally, subgroup analysis was limited to Tai Chi, Baduanjin, and their matching single intervention durations because all studies had intervention cycles and frequencies of 12 weeks and 3 times per week. Both Tai Chi (MD = 0.11, 95% CI: 0.07–0.16, *p* < 0.001) and Baduanjin (MD = 0.14, 95% CI: 0.08–0.20, *p* < 0.001) improved 6m_GS, according to the results (Table 2), with no significant difference between subgroups (*p* = 0.50). Session lengths of ≤ 40 min and > 40 min were associated with increases in 6m_GS (*p* ≤ 0.01).

#### 3.4.3 Appendicular skeletal muscle mass index

**Figure 7:**
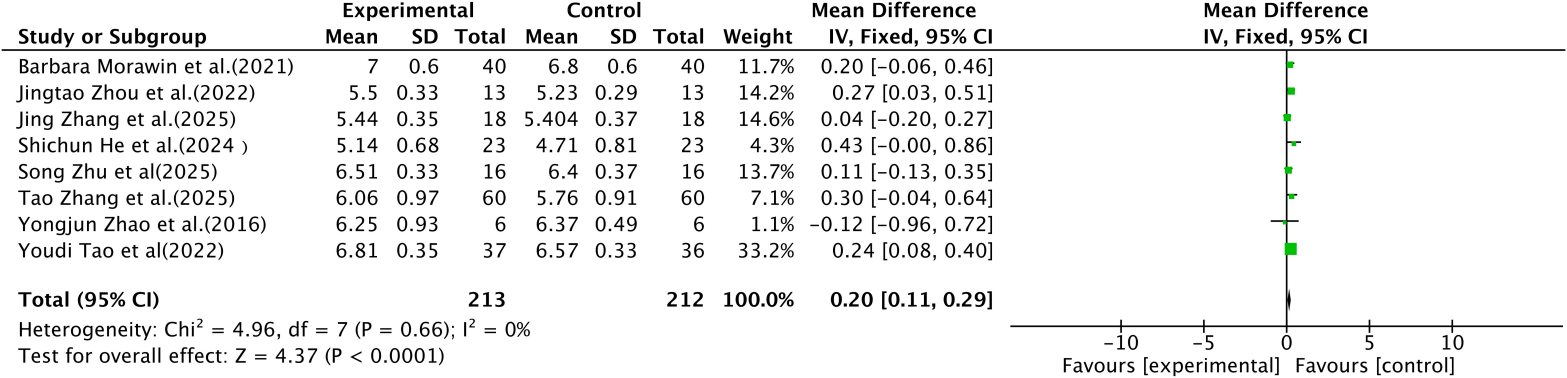
Forest plot of the effects of traditional Chinese exercises on appendicular skeletal muscle mass index in older adults with sarcopenia.

A total of 8 RCTs, comprising 425 participants (213 in the experimental group and 212 in the control group) (22, 27–30, 32, 33, 35) reported the effects of TCEs on ASMI in older adults with sarcopenia. The studies' modest heterogeneity (*I*^2^ = 0%, *P* = 0.66) supported the application of a fixed-effects model. TCEs improved ASMI in this cohort, according to the pooled results (MD = 0.20, 95% CI: 0.11–0.29, *p* < 0.001) (Figure 7, Supplementary Figure S7). Sensitivity analyses suggested that the findings were robust and not materially altered (Supplementary Figures S13, S13.1, S13.2). Egger's test showed that there was no publication bias (*p* = 0.903 > 0.05). Given the same intervention durations and frequencies across studies, subgroup analyses were conducted for exercise type and session duration. The findings demonstrated that Tai Chi increased ASMI (MD = 0.25, 95% CI: 0.12–0.38, *p* < 0.01), whereas Yi Jin Jing (MD = 0.24, 95% CI:−0.07–0.55, *p* = 0.13) and Baduanjin (MD = 0.14, 95% CI: 0.00–0.28, *p* = 0.05) (Table 2). There was no significant difference between the two subgroups when it came to session time; both ≤ 40 min and > 40 min were linked to substantial improvements in ASMI.

#### 3.4.4 Timed up-and-go test

**Figure 8:**
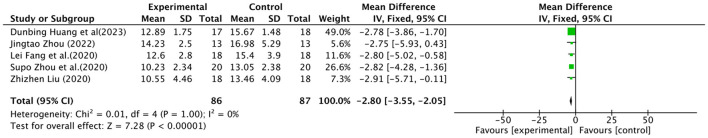
Forest plot of the effects of traditional Chinese exercises on the timed up-and-go test in older adults with sarcopenia.

A total of 5 RCTs, comprising 173 participants (86 in the experimental group and 87 in the control group) (24, 30, 31, 35, 36) reported the effects of TCEs on TUGT performance in older adults with sarcopenia. The studies' modest heterogeneity (*I*^2^ = 0%, *P* = 1.00) supported the application of a fixed-effects model. TCEs improved TUGT in this cohort, according to the pooled results (MD = −2.80, 95% CI: −3.55–−2.05, *p* < 0.001) (Figure 8, Supplementary Figure S8). Sensitivity analyses suggested that the findings were robust and not materially altered (Supplementary Figures S14, S14.1). Egger's test showed that there was no publication bias (*p* = 0.503 > 0.05). Only session duration was examined since subgroup analyses for intervention duration or frequency were not possible due to data constraints (Table 2). The findings demonstrated that there was no significant difference between the subgroups and that both ≤ 40 min and > 40 min were linked to substantial increases in TUGT performance.

#### 3.4.5 Sit-to-stand tests

**Figure 9:**
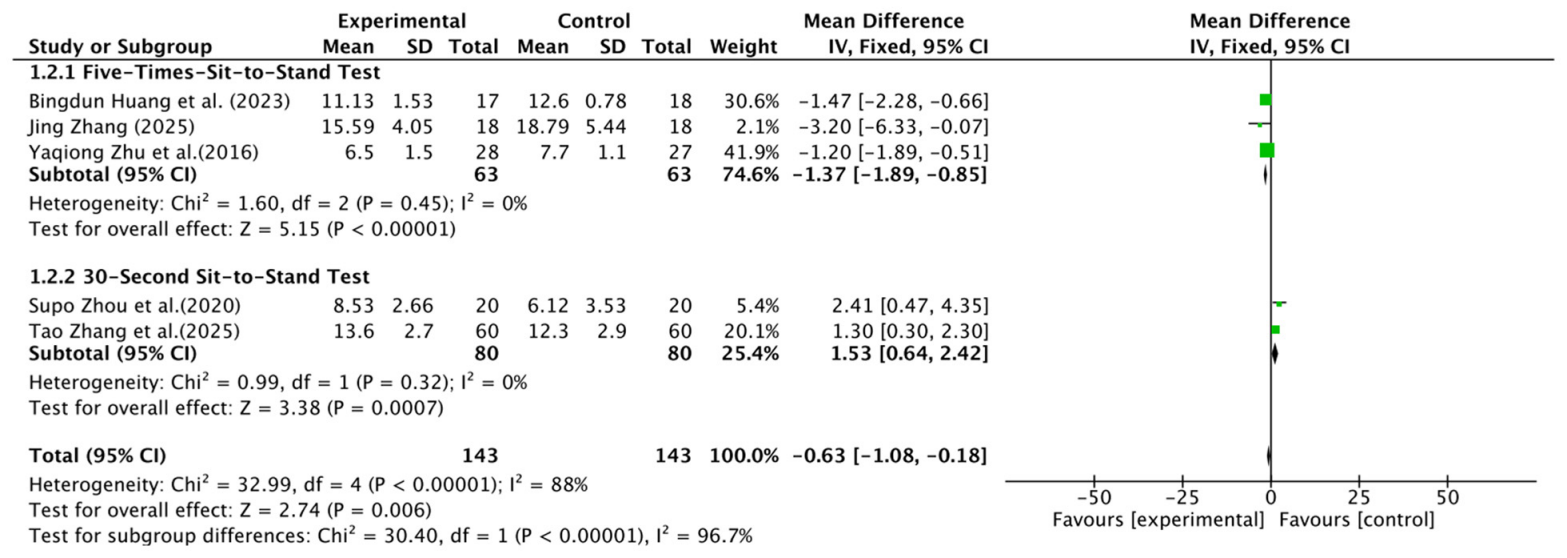
Forest plot of the effects of traditional Chinese exercises on the sit-to-stand test in older adults with sarcopenia.

A total of 5 RCTs reported the effects of TCEs on sit-to-stand tests in older adults with sarcopenia, comprising 286 participants (143 in the experimental group and 143 in the control group) (27, 28, 31, 36, 37). Subgroup analyses were carried out for the FTSST and 30s STS due to the variation in sit-to-stand test methods between studies (Figure 9, Supplementary Figure S9). 3 studies were included for FTSST. The studies' low heterogeneity (*I*^2^ = 0%, *P* = 0.45) justified the adoption of a fixed-effects model. The pooled data showed that TCEs improved FTSST performance in this cohort (MD = −1.37, 95% CI:−1.89–−0.85, *p* < 0.001). Sensitivity analyses suggested that the findings were robust and not materially altered (Supplementary Figure S15). Egger's test showed no indication of publication bias (*p* = 0.152 > 0.05). 2 studies were considered for the 30s STS, and a fixed-effects model (*I*^2^ = 0%, *P* = 0.32) was used. TCEs enhanced 30s STS performance, according to the data (MD = 1.53, 95% CI: 0.64–2.42, *p* < 0.001) (Table 2).

#### 3.4.6 Short physical performance battery

**Figure 10:**
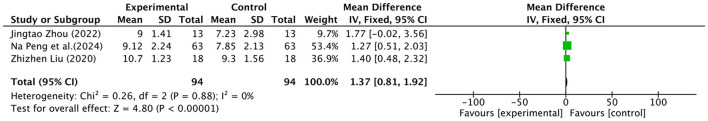
Forest plot of the effects of traditional Chinese exercises on the short physical performance battery in older adults with sarcopenia.

A total of 3 RCTs reported the effects of TCEs on SPPB scores in older adults with sarcopenia, comprising 188 participants (94 in the experimental group and 94 in the control group) (24, 25, 30). The studies' minimal heterogeneity (*I*^2^ = 0%, *P* = 0.88) supported the application of a fixed-effects model. TCEs raised SPPB scores in this cohort, according to the meta-analysis results (MD = 1.37, 95% CI: 0.81–1.92, *p* < 0.001) (Figure 10, Supplementary Figure S10). Sensitivity analyses suggested that the findings were robust and not materially altered (Supplementary Figure S16). No indication of publication bias was found using Egger's test (*p* = 0.111 > 0.05). No additional subgroup analyses were carried out since just three studies used SPPB as an outcome measure.

#### 3.4.7 Certainty of evidence

The certainty of evidence for each outcome in this review was assessed using the GRADE approach. Overall, the certainty of evidence ranged from low to moderate. HS was rated as moderate certainty, while most other outcomes, including 6m_GS,

## 4 Discussion

Muscle strength, muscle mass, and physical function are key components in the assessment and management of sarcopenia, yet effective and sustainable interventions remain limited. This systematic review and meta-analysis synthesized evidence from 17 RCTs to evaluate the effects of TCEs on these outcomes in older adults with sarcopenia.

HS is widely recognized as a simple and reliable indicator of global muscle strength and is associated with adverse clinical outcomes in older adults. In the present study, TCEs were associated with an average increase of 1.13 kg in HS. This magnitude of improvement falls within previously reported minimal clinically important difference (MCID) ranges for older adults (approximately 1.0–1.5 kg) (38). Furthermore, prior evidence has demonstrated that each 1 kg increase in HS may be associated with a 3%-5% reduction in all-cause mortality among older adults (39). Standardized measurement and interpretation of HS have been well established in clinical and epidemiological studies (40). Nevertheless, interpretation should remain cautious given variability in intervention protocols, participant characteristics, and outcome measurement methods across studies. Subgroup analyses suggested potential differences between exercise methods and session durations; however, these findings should be regarded as exploratory and hypothesis-generating, particularly given limited study numbers and clinical heterogeneity.

Gait speed is a well-established indicator of functional status in older adults and is often considered a key marker of frailty and health outcomes (41). In this study, TCEs, particularly Tai Chi and Baduanjin, were associated with improvements in 6m_GS. These exercise forms emphasize postural control, balance, and coordinated movement, which may contribute to improved mobility. Tai Chi has been shown to enhance postural stability and balance performance in older adults and clinical populations (42), while Baduanjin and related Qigong exercises have demonstrated beneficial effects on muscle function in sarcopenic populations (43). However, these mechanistic interpretations remain indirect and were not directly examined in the included trials. In addition, clinically meaningful change thresholds for gait speed (approximately 0.1 m/s in older adults) should be considered when interpreting these findings. Given the limited number of studies and variability in intervention design, comparative conclusions across different TCEs should be interpreted cautiously.

ASMI represents an important marker of muscle quantity and sarcopenia severity. In this analysis, TCEs were associated with a modest increase in ASMI (0.20 kg/m^2^). While reductions in muscle mass are clinically relevant in aging populations, even low levels of muscle loss have been associated with adverse outcomes in older adults (44). Age-related declines in muscle size and strength are well documented and contribute to sarcopenia progression (45). However, established minimal clinically important difference thresholds for ASMI remain insufficiently defined, limiting definitive interpretation of clinical relevance. Previous consensus and position statements emphasize the importance of combining muscle mass and function in clinical assessment (46). Subgroup analyses did not demonstrate significant differences between exercise methods or session duration, consistent with prior meta-analyses of TCEs (47, 48), suggesting that overall training exposure may be more relevant than specific exercise type; however, this interpretation remains speculative.

Functional performance outcomes, including TUGT, sit-to-stand tests, and SPPB, further supported potential benefits of TCEs on mobility and lower limb function. TUGT and related functional tests are widely used to assess mobility limitation and fall risk in older adults (49, 50). Sit-to-stand performance reflects lower limb strength and functional independence, with standardized protocols and prognostic value increasingly recognized in older adults (51, 52). Functional performance measures are strongly associated with fall risk and mobility limitation in older adults (53). In the present study, the observed improvement in SPPB score (1.37 points) may be clinically meaningful, as SPPB is strongly associated with mortality and functional decline (54), and a 1-point change has been suggested to represent a relevant clinical difference in at-risk older adults (55). Nevertheless, due to limited sample sizes and substantial clinical heterogeneity across interventions, populations, and outcome measures, these findings should be interpreted with caution.

It is important to note that nearly all included studies were conducted in China, with only one study originating from another country. This geographic concentration may limit the external validity and generalizability of the findings. Cultural familiarity with TCEs, differences in healthcare systems, and variations in participant expectations may influence adherence, engagement, and perceived effectiveness. Accordingly, caution is warranted when extrapolating these findings to non-Chinese or more diverse populations.

Overall, while TCEs appear to be associated with potential improvements in muscle strength, muscle mass, and physical function in older adults with sarcopenia, the certainty of the evidence remains limited. Subgroup findings should be interpreted as exploratory and hypothesis-generating rather than confirmatory, and should not be considered evidence for the superiority of specific exercise modalities, training durations, or dosages.

## 5 Conclusion

This systematic review and meta-analysis suggests that TCEs may be associated with improvements in muscle strength, muscle mass, and physical function in older adults with sarcopenia. The certainty of evidence was rated as low to moderate according to GRADE, which limits confidence in the magnitude of these effects. Findings from subgroup analyses related to exercise modality and training characteristics should be interpreted cautiously, as they were based on a small number of studies and are better considered exploratory. Overall, while TCEs may offer a possible non-pharmacological approach for sarcopenia, the current evidence is not sufficient to support firm conclusions regarding their effectiveness or optimal exercise prescription. Well-designed, large-scale RCTs with broader population representation are still needed to clarify these effects and strengthen the evidence base.

## 6 Limitations

Several limitations of the present study should be acknowledged. (1) Although subgroup analyses were conducted to explore potential differences in exercise modality and training characteristics, the number of studies within each subgroup was small. Therefore, these findings should be interpreted as exploratory and hypothesis-generating rather than providing evidence for optimal intervention parameters. (2) Potential clinical heterogeneity may exist across the included trials. Although statistical heterogeneity was low for several outcomes, variations in exercise modality, intervention dose (frequency, duration, and session length), participant characteristics, and control conditions may have contributed to underlying clinical heterogeneity and should be considered when interpreting the pooled estimates. (3) The certainty of evidence was generally low to moderate according to the GRADE assessment, mainly due to risk of bias, imprecision, and small sample sizes, which may further reduces confidence in the robustness of the findings. (4) The evidence base was geographically concentrated, with nearly all included studies conducted in China and only one study from another country. This limits the generalizability of the findings to other healthcare systems and populations.

## Data Availability

The original contributions presented in the study are included in the article/Supplementary material. Further inquiries can be directed to the corresponding authors.

## Supplementary material

Supplementary File S1 PRISMA checklist.

Supplementary File S2 PROSPERO protocol.

Supplementary File S3 Search strategy of PubMed.

Supplementary File S4 Full-text screening and datasets.

Supplementary File S5 Certainty of evidence.

Supplementary Figure S1 PRISMA flow diagram of the study selection.

Supplementary Figure S2 RoB2 risk of bias ratings for each eligible study.

Supplementary Figure S3 The assessment of each risk of bias item in each study included in the risk of bias summary.

Supplementary Figure S4 Number of included studies corresponding to each primary outcome indicator.

Supplementary Figure S5 Forest plot of handgrip strength.

Supplementary Figure S5.1 Forest plot of handgrip strength for group subgroup analyses (exercise methods).

Supplementary Figure S5.2 Forest plot of handgrip strength for group subgroup analyses (length, weeks).

Supplementary Figure S5.3 Forest plot of handgrip strength for group subgroup analyses (frequency, sessions/week).

Supplementary Figure S5.4 Forest plot of handgrip strength for group subgroup analyses (session duration, minutes).

Supplementary Figure S6 Forest plot of 6-meter walk test.

Supplementary Figure S6.1 Forest plot of 6-Meter walk test for group subgroup analyses (exercise methods).

Supplementary Figure S6.2 Forest plot of 6-Meter walk test for group subgroup analyses (frequency, sessions/week).

Supplementary Figure S6.3 Forest plot of 6-Meter walk test for group subgroup analyses (Session duration, minutes).

Supplementary Figure S7 Forest plot of appendicular skeletal muscle mass index.

Supplementary Figure S7.1 Forest plot of appendicular skeletal muscle mass index for group subgroup analyses (exercise methods).

Supplementary Figure S7.2 Forest plot of appendicular skeletal muscle mass index for group subgroup analyses (Session duration, minutes).

Supplementary Figure S8 Forest plot of timed up and go test.

Supplementary Figure S8.1 Forest plot of timed up and go test for group subgroup analyses (Session duration, minutes).

Supplementary Figure S9 Forest plot of sit-to-stand test.

Supplementary Figure S10 Forest plot of short physical performance battery.

Supplementary Figure S11 Random-effects forest plot of grip strength.

Supplementary Figure S11.1 Random-effects forest plot of handgrip strength for group subgroup analyses (exercise methods).

Supplementary Figure S11.2 Random-effects forest plot of handgrip strength for group subgroup analyses (length, weeks).

Supplementary Figure S11.3 Random-effects forest plot of handgrip strength for group subgroup analyses (frequency, sessions/week).

Supplementary Figure S11.4 Random-effects forest plot of handgrip strength for group subgroup analyses (session duration, minutes).

Supplementary Figure S12 Random-effects forest plot of 6-meter walk test.

Supplementary Figure S12.1 Random-effects forest plot of 6-Meter walk test for group subgroup analyses (exercise methods).

Supplementary Figure S12.2 Random-effects forest plot of 6-Meter walk test for group subgroup analyses (frequency, sessions/week).

Supplementary Figure S12.3 Random-effects forest plot of 6-Meter walk test for group subgroup analyses (Session duration, minutes).

Supplementary Figure S13 Random-effects forest plot of appendicular skeletal muscle mass index.

Supplementary Figure S13.1 Random-effects forest plot of appendicular skeletal muscle mass index for group subgroup analyses (exercise methods).

Supplementary Figure S13.2 Random-effects forest plot of appendicular skeletal muscle mass index for group subgroup analyses (Session duration, minutes).

Supplementary Figure S14 Random-effects forest plot of timed up and go test.

Supplementary Figure S14.1 Random-effects forest plot of timed up and go test for group subgroup analyses (Session duration, minutes).

Supplementary Figure S15 Random-effects forest plot of sit-to-stand test.

Supplementary Figure S16 Random-effects forest plot of short physical performance battery.
