# Supplementary material for: Traditional Chinese exercises on muscle strength, muscle mass, and physical function in older adults with sarcopenia: a systematic review and meta-analysis
Source: Front Med (Lausanne). 2026 Aug 5;13:1841875. doi: 10.3389/fmed.2026.1841875 (PMC13487372; doi:10.3389/fmed.2026.1841875)
Supplement: Supplementary file 1 [file Data_Sheet_2.pdf]

NIHR | National Institute for Health and Care Research

PROSPERO
International prospective register of systematic reviews

Traditional Chinese Exercise Interventions for Sarcopenia and Its Comorbidities in the Elderly: A Meta-Analysis and Systematic Review

Ruidie Ma, Wang Zhu, Si Ma, Yingbing Mei, Yu Guo

REVIEW TITLE AND BASIC DETAILS

Review title

Traditional Chinese Exercise Interventions for Sarcopenia and Its Comorbidities in the Elderly: A Meta-Analysis and Systematic Review

Condition or domain being studied

Therapeutic Exercise

Rationale for the review

This systematic review and meta-analysis will evaluate the effectiveness of Traditional Chinese Exercise (TCE), including Tai Chi, Baduanjin, on muscle strength, muscle mass, and physical function in older adults (aged ⩾60 years) diagnosed with sarcopenia and its associated complications, such as frailty and osteoporosis. The review will include randomized controlled trials (RCTs) comparing TCE interventions to inactive controls (e.g., usual care, no treatment, or health education). Primary outcomes will focus on changes in handgrip strength, appendicular skeletal muscle mass index (ASMI), and gait speed, with secondary outcomes including fall incidence and quality of life. This synthesis aims to provide evidence-based insights into the role of traditional Chinese exercise as a potential therapeutic strategy for managing age-related muscle degeneration.

Review objectives

Exploring the effects of traditional Chinese exercise on muscle strength, mass, and physical function in elderly sarcopenia through meta-analysis

Keywords

Traditional Chinese exercises; Sarcopenia in the elderly; Meta-analysis

Country

China

ELIGIBILITY CRITERIA

Population

Included
Patients with sarcopenia (age> 60 years)

Excluded
non–human subjects, intervention subjects with an average age <60 years, or does not mention diagnostic criteria or guidelines for sarcopenia

Intervention(s) or exposure(s)

Included
Physical Exercises; Tai Chi

Comparator(s) or control(s)

Included
PICO tags selected: Usual Care

Study design
Only randomized study types will be included.

Context
Studies in hospital accident and emergency departments

TIMELINE OF THE REVIEW

Date of first submission to PROSPERO
17 March 2026

Review timeline
Start date: 18 March 2026. End date: 17 September 2026.

Date of registration in PROSPERO
17 March 2026

AVAILABILITY OF FULL PROTOCOL

Availability of full protocol
A full protocol has not been written.

SEARCHING AND SCREENING

Search for unpublished studies
Only unpublished studies will be sought.

Main sources that will be searched
The main databases to be searched are *CLIB – The Cochrane Library*, *Embase – Embase via Ovid*, *PubMed* and *SCI – Science Citation Index*.

Search language restrictions
There are no language restrictions.

Search date restrictions
There are no search date restrictions.

Other methods of identifying studies
Other studies will be identified by: *contacting authors or experts*.

Link to search strategy
A full search strategy is not available.

Selection process
Studies will be screened independently by at least two people (or person/machine combination) with a process to resolve differences.

Other relevant information about searching and screening
None

DATA COLLECTION PROCESS

Data extraction from published articles and reports
Data will be extracted independently by at least two people (or person/machine combination) with a process to resolve differences.

Authors will be asked to provide any required data not available in published reports.

Study risk of bias or quality assessment
Risk of bias will be assessed using: *Cochrane RoB-1*

Data will be assessed independently by at least two people (or person/machine combination) with a process to resolve differences.

Additional information will be sought from study investigators if required information is unclear or unavailable in the study publications/reports.

Reporting bias assessment
Risk of bias due to missing results will be assessed

Certainty assessment
We will use GRADE to evaluate the indicators.

OUTCOMES TO BE ANALYSED

Main outcomes
Grip strength;Skeletal Muscle Mass Index;Sit-to-stand test;Stand-Walk Test

Additional outcomes
Muscle strength

PLANNED DATA SYNTHESIS

Strategy for data synthesis

For time–to–event outcomes such as overall survival and disease–free survival, data will be combined using the generic inverse variance method to calculate pooled hazard ratios (HRs) with 95% confidence intervals. Statistical heterogeneity will be assessed using the I^2 statistic; if I^2 is less than 50%, a fixed–effects model will be applied, whereas a random–effects model will be used if heterogeneity is moderate to high ($I^2 \geqslant 50\%$). Where sufficient data are available, subgroup analyses will be conducted based on clinical stage and chemotherapy regimen, and sensitivity analyses will be performed by excluding studies at high risk of bias to test the robustness of the findings.

CURRENT REVIEW STAGE

Stage of the review at this submission

Review stage	Started	Completed
Pilot work	✓	
Formal searching/study identification	✓	
Screening search results against inclusion criteria		
Data extraction or receipt of IPD		
Risk of bias/quality assessment		
Data synthesis		

Review status

The review is currently planned or ongoing.

Publication of review results

Results of the review will be published in English.

REVIEW AFFILIATION, FUNDING AND PEER REVIEW

Review team members

Miss Ruidie Ma (review guarantor). ORCID: 0009–0001–6400–3353. Hubei University of Traditional Chinese Medicine. China.

No conflict of interest declared.

Mr Wang Zhu. Hubei Provincial Hospital of Traditional Chinese Medicine. China.

No conflict of interest declared.

Mr Si Ma. Yuntianhua Co., Ltd. Formaldehyde Division Shuifu Branch. China.

No conflict of interest declared.

Mr Yingbing Mei. Hubei Provincial Hospital of Traditional Chinese Medicine. China.

No conflict of interest declared.

Mr Yu Guo. Hubei University of Traditional Chinese Medicine. China.

No conflict of interest declared.

Named contact

Mr Yingbing Mei (myb114@163.com). Hubei Provincial Hospital of Traditional Chinese Medicine. China.

Review affiliation

Hubei University of Traditional Chinese Medicine

Funding source

Review has no funding and no agreed support from an academic institution and is done in authors' own time.

Peer review

There has been no peer review of this planned review.

ADDITIONAL INFORMATION

Review conflict of interest

Declared individual interests are recorded under team member details.. No additional interests are recorded for this review.

Medical Subject Headings

Aged; China; Exercise Therapy; Humans; Muscle Strength; Sarcopenia

SIMILAR REVIEWS

Check for similar records already in PROSPERO

PROSPERO identified a number of existing PROSPERO records that were similar to this one (last check made on 17 March 2026). These are shown below along with the reasons given by that the review team for the reviews being different and/or proceeding.

- Analysis of Exercise Intervention on Elderly Patients with Sarcopenia: A Meta-Analysis [published 13 November 2025] [CRD420251182278]. The review was judged **not to be similar**
- A Network Meta-Analysis of Traditional Chinese Medicine for the Treatment of Sarcopenia [published 8 January 2025] [CRD42025623875]. The review was judged **not to be similar**
- SYSTEMATIC REVIEW AND META-ANALYSIS OF THE EFFECTS OF EXERCISE FOR ELDERLY PEOPLE WITH SARCOPENIA. [published 29 May 2025] [CRD420251062085]. The review was judged **not to be similar**

PROSPERO version history

- Version 1.0, published 17 Mar 2026

Disclaimer

The content of this record displays the information provided by the review team. PROSPERO does not peer review registration records or endorse their content.

PROSPERO accepts and posts the information provided in good faith; responsibility for record content rests with the review team. The guarantor for this record has affirmed that

the information provided is truthful and that they understand that deliberate provision of inaccurate information may be construed as scientific misconduct.

PROSPERO does not accept any liability for the content provided in this record or for its use. Readers use the information provided in this record at their own risk.

Any enquiries about the record should be referred to the named review contact
