# Supplementary material for: Traditional Chinese exercises on muscle strength, muscle mass, and physical function in older adults with sarcopenia: a systematic review and meta-analysis
Source: Front Med (Lausanne). 2026 Aug 5;13:1841875. doi: 10.3389/fmed.2026.1841875 (PMC13487372; doi:10.3389/fmed.2026.1841875)
Supplement: Supplementary file 2 [file Supplementary_file_1.pdf]

S1 Fig. PRISMA flow diagram of the study selection.

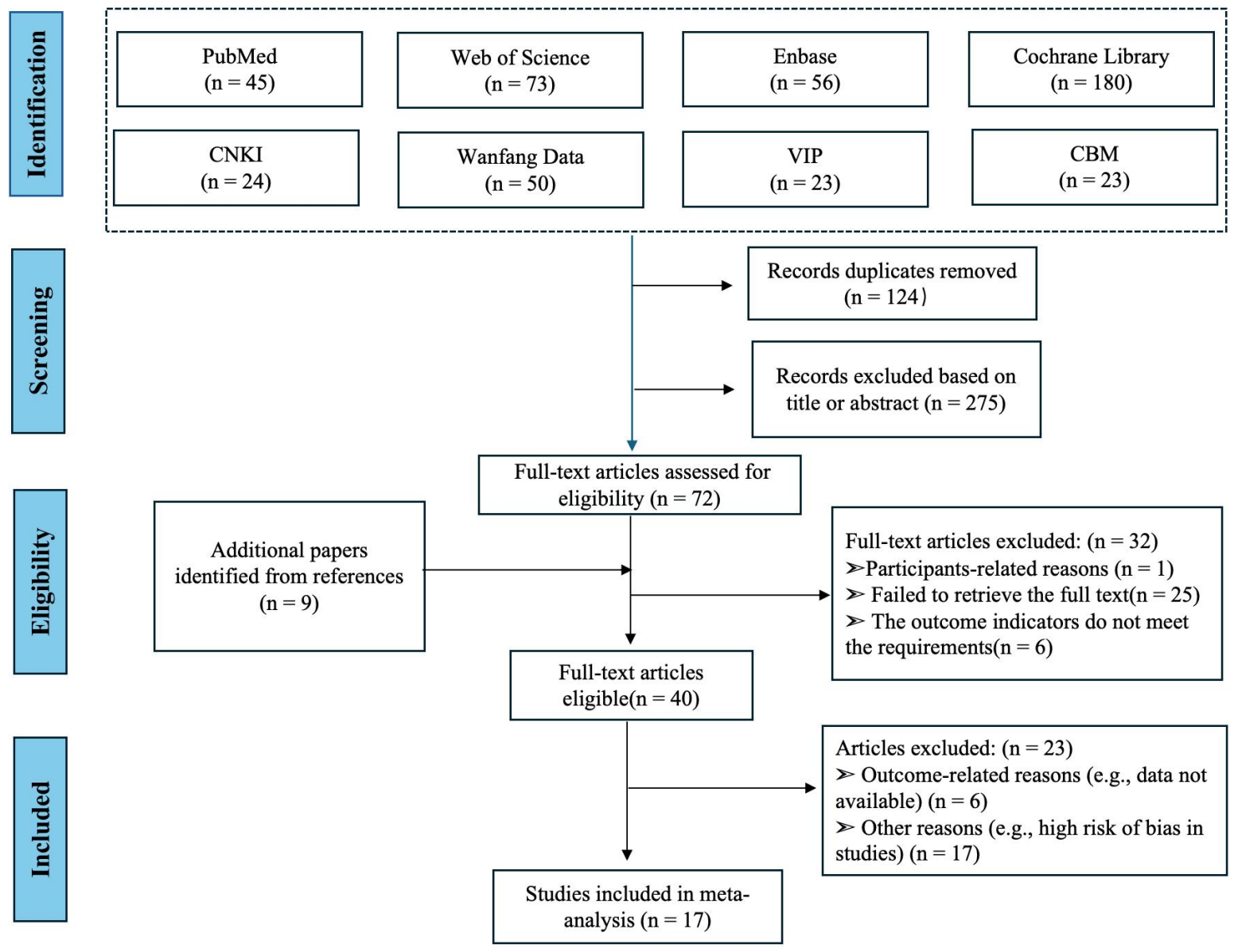

S2 Fig. RoB2 risk of bias ratings for each eligible study.

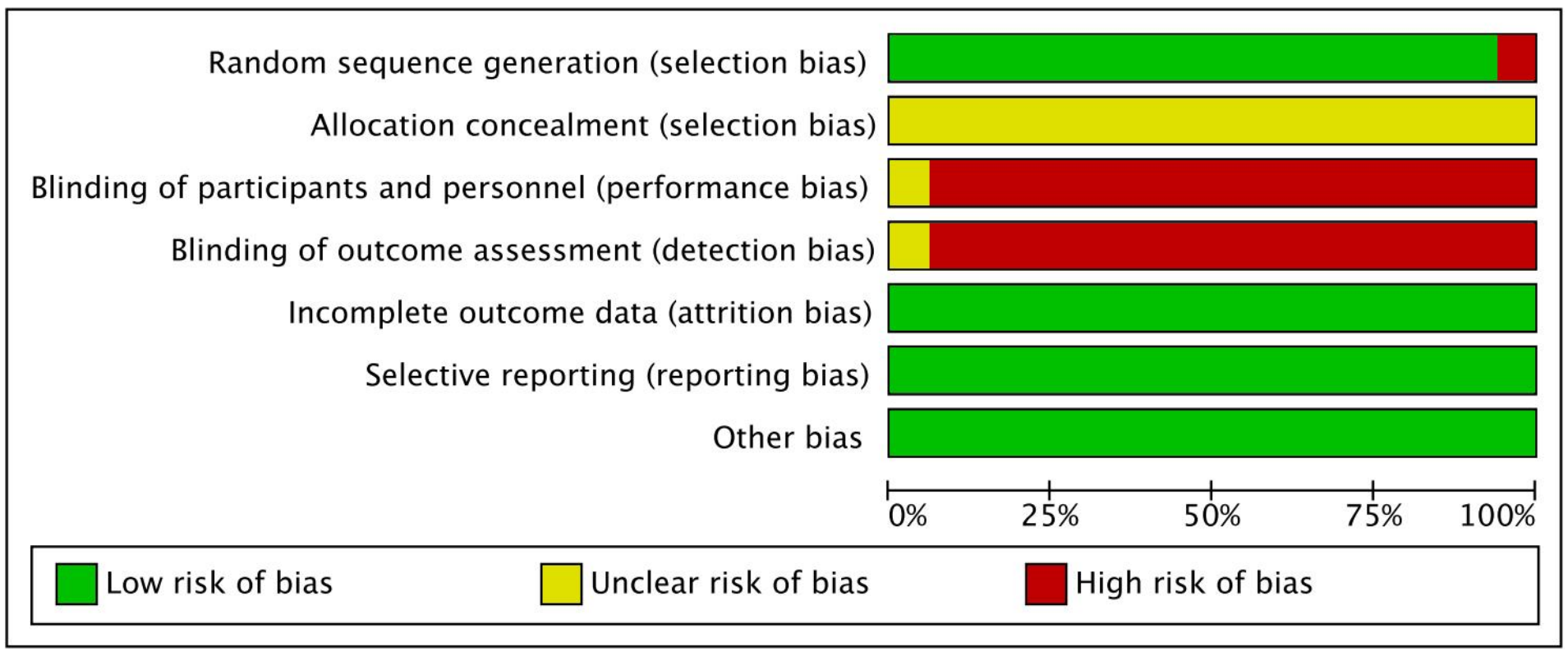

S3 Fig. The assessment of each risk of bias item in each study included in the risk of bias summary.

	Random sequence generation (selection bias)	Allocation concealment (selection bias)	Blinding of participants and personnel (performance bias)	Blinding of outcome assessment (detection bias)	Incomplete outcome data (attrition bias)	Selective reporting (reporting bias)	Other bias
Barbara Morawin et al.(2021)	+	?	?	?	+	+	+
Dunbing Huang et al.(2023)	+	?	-	-	+	+	+
Jingtao Zhou (2022)	-	?	-	-	+	+	+
Jing Zhang (2025)	+	?	-	-	+	+	+
Lei Fang et al.(2020)	+	?	-	-	+	+	+
Na Peng et al.(2024)	+	?	-	-	+	+	+
Shichun He (2024)	+	?	-	-	+	+	+
Song Zhu (2025)	+	?	-	-	+	+	+
Supo Zhou et al.(2020)	+	?	-	-	+	+	+
Tao Zhang et al.(2025)	+	?	-	-	+	+	+
Xijing Liu et al.(2024)	+	?	-	-	+	+	+
Xuechen Xu et al.(2022)	+	?	-	-	+	+	+
Yaqiong Zhu et al.(2016)	+	?	-	-	+	+	+
Yaqiong Zhu et al.(2019)	+	?	-	-	+	+	+
Yongjun Zhao et al.(2016)	+	?	-	-	+	+	+
Youdi Tao et al.(2022)	+	?	-	-	+	+	+
Zhizhen Liu (2020)	+	?	-	-	+	+	+

S4 Fig. Number of included studies corresponding to each primary outcome indicator.

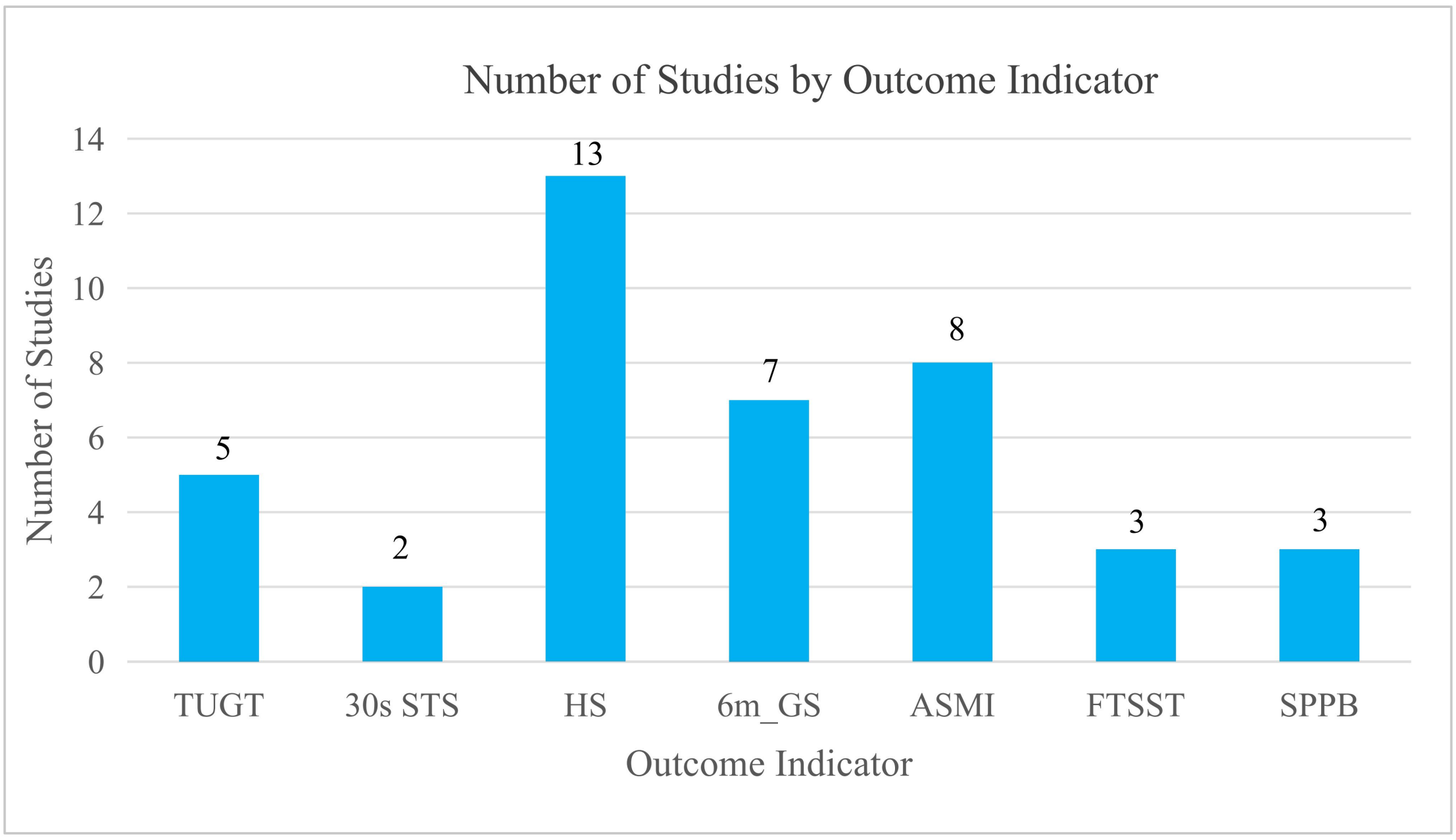
Number of Studies by Outcome Indicator
Number of Studies
14
12
10
8
6
4
2
0
5
2
13
7
8
3
3
TUGT
30s STS
HS
6m_GS
ASMI
FTSST
SPPB
Outcome Indicator
