# Supplementary material for: Traditional Chinese exercises on muscle strength, muscle mass, and physical function in older adults with sarcopenia: a systematic review and meta-analysis
Source: Front Med (Lausanne). 2026 Aug 5;13:1841875. doi: 10.3389/fmed.2026.1841875 (PMC13487372; doi:10.3389/fmed.2026.1841875)

S14 Fig. Random-effects forest plot of timed up and go test.

Study or Subgroup	Experimental Mean	Experimental SD	Experimental Total	Control Mean	Control SD	Control Total	Weight	Mean Difference IV, Random, 95% CI
Dunbing Huang et al(2023)	12.89	1.75	17	15.67	1.48	18	49.0%	−2.78 [−3.86, −1.70]
Jingtao Zhou (2022)	14.23	2.5	13	16.98	5.29	13	5.6%	−2.75 [−5.93, 0.43]
Lei Fang et al.(2020)	12.6	2.8	18	15.4	3.9	18	11.6%	−2.80 [−5.02, −0.58]
Supo Zhou et al.(2020)	10.23	2.34	20	13.05	2.38	20	26.6%	−2.82 [−4.28, −1.36]
Zhizhen Liu (2020)	10.55	4.46	18	13.46	4.09	18	7.3%	−2.91 [−5.71, −0.11]
**Total (95% CI)**			**86**			**87**	**100.0%**	**−2.80 [−3.55, −2.05]**

Heterogeneity: $Tau^2 = 0.00$; $Chi^2 = 0.01$, df = 4 (P = 1.00); $I^2 = 0\%$
Test for overall effect: Z = 7.28 (P < 0.00001)

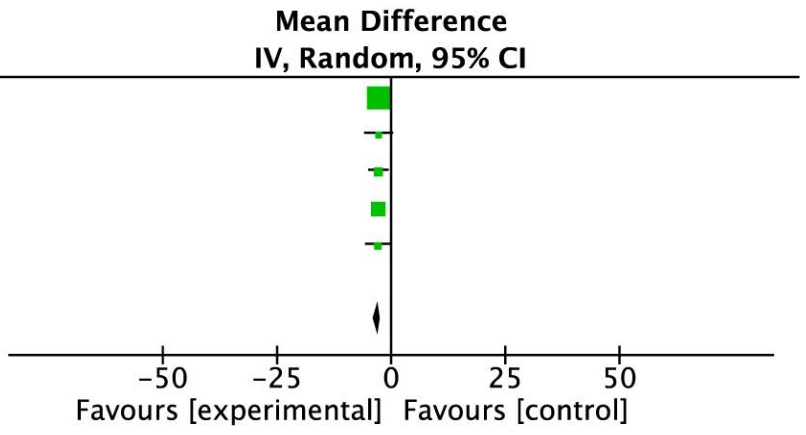

S14.1 Fig. Random-effects forest plot of timed up and go test for group subgroup analyses (Session duration, minutes).

Study or Subgroup	Experimental Mean	Experimental SD	Experimental Total	Control Mean	Control SD	Control Total	Weight	Mean Difference IV, Random, 95% CI
**1.2.1 ≤ 40**								
Lei Fang et al.(2020)	12.6	2.8	18	15.4	3.9	18	11.6%	−2.80 [−5.02, −0.58]
Supo Zhou et al.(2020)	10.23	2.34	20	13.05	2.38	20	26.6%	−2.82 [−4.28, −1.36]
**Subtotal (95% CI)**			**38**			**38**	**38.1%**	**−2.81 [−4.04, −1.59]**
Heterogeneity: $Tau^2 = 0.00$; $Chi^2 = 0.00$, df = 1 (P = 0.99); $I^2 = 0\%$								
Test for overall effect: Z = 4.52 (P < 0.00001)								
**1.2.2 > 40**								
Dunbing Huang et al(2023)	12.89	1.75	17	15.67	1.48	18	49.0%	−2.78 [−3.86, −1.70]
Jingtao Zhou (2022)	14.23	2.5	13	16.98	5.29	13	5.6%	−2.75 [−5.93, 0.43]
Zhizhen Liu (2020)	10.55	4.46	18	13.46	4.09	18	7.3%	−2.91 [−5.71, −0.11]
**Subtotal (95% CI)**			**48**			**49**	**61.9%**	**−2.79 [−3.75, −1.83]**
Heterogeneity: $Tau^2 = 0.00$; $Chi^2 = 0.01$, df = 2 (P = 1.00); $I^2 = 0\%$								
Test for overall effect: Z = 5.71 (P < 0.00001)								
**Total (95% CI)**			**86**			**87**	**100.0%**	**−2.80 [−3.55, −2.05]**

Heterogeneity: $Tau^2 = 0.00$; $Chi^2 = 0.01$, df = 4 (P = 1.00); $I^2 = 0\%$
Test for overall effect: Z = 7.28 (P < 0.00001)
Test for subgroup differences: $Chi^2 = 0.00$, df = 1 (P = 0.98), $I^2 = 0\%$

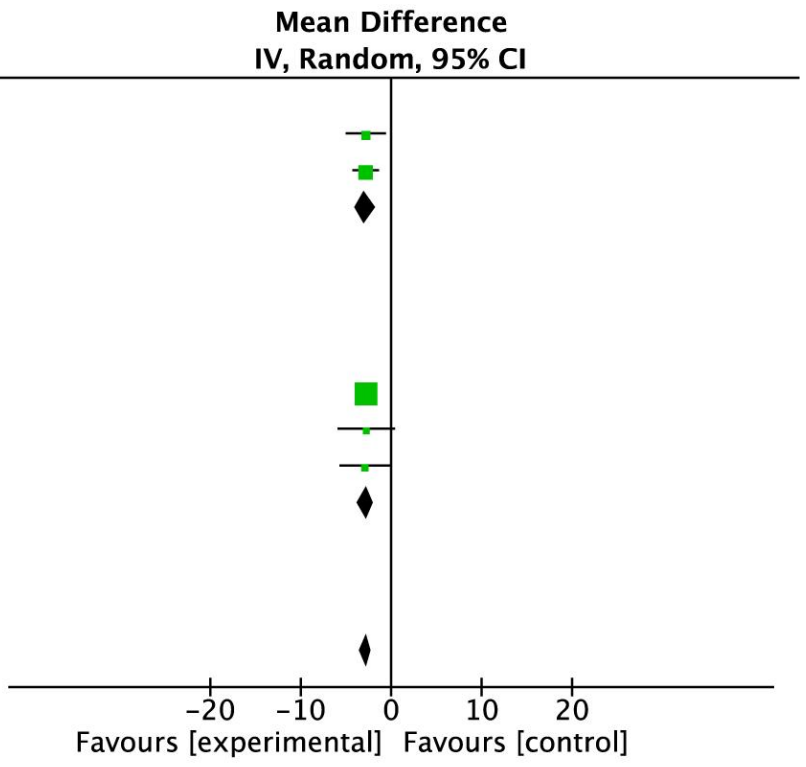

Supplement: Supplementary file 3 [file Supplementary_file_10.pdf]
