# Supplementary material for: Traditional Chinese exercises on muscle strength, muscle mass, and physical function in older adults with sarcopenia: a systematic review and meta-analysis
Source: Front Med (Lausanne). 2026 Aug 5;13:1841875. doi: 10.3389/fmed.2026.1841875 (PMC13487372; doi:10.3389/fmed.2026.1841875)

S15 Fig. Random-effects forest plot of sit-to-stand test.

Study or Subgroup	Experimental Mean	Experimental SD	Experimental Total	Control Mean	Control SD	Control Total	Weight	Mean Difference IV, Random, 95% CI
**1.2.1 Five-Times-Sit-to-Stand Test**								
Bingdun Huang et al. (2023)	11.13	1.53	17	12.6	0.78	18	23.6%	−1.47 [−2.28, −0.66]
Jing Zhang (2025)	15.59	4.05	18	18.79	5.44	18	11.8%	−3.20 [−6.33, −0.07]
Yaqiong Zhu et al.(2016)	6.5	1.5	28	7.7	1.1	27	24.1%	−1.20 [−1.89, −0.51]
**Subtotal (95% CI)**			**63**			**63**	**59.6%**	**−1.37 [−1.89, −0.85]**
Heterogeneity: $Tau^2 = 0.00$; $Chi^2 = 1.60$, df = 2 (P = 0.45); $I^2 = 0\%$								
Test for overall effect: Z = 5.15 (P < 0.00001)								
**1.2.2 30-Second Sit-to-Stand Test**								
Supo Zhou et al.(2020)	8.53	2.66	20	6.12	3.53	20	17.7%	2.41 [0.47, 4.35]
Tao Zhang et al.(2025)	13.6	2.7	60	12.3	2.9	60	22.8%	1.30 [0.30, 2.30]
**Subtotal (95% CI)**			**80**			**80**	**40.4%**	**1.53 [0.64, 2.42]**
Heterogeneity: $Tau^2 = 0.00$; $Chi^2 = 0.99$, df = 1 (P = 0.32); $I^2 = 0\%$								
Test for overall effect: Z = 3.38 (P = 0.0007)								
**Total (95% CI)**			**143**			**143**	**100.0%**	**−0.29 [−1.76, 1.18]**

Heterogeneity: $Tau^2 = 2.21$; $Chi^2 = 32.99$, df = 4 (P < 0.00001); $I^2 = 88\%$
Test for overall effect: Z = 0.39 (P = 0.70)
Test for subgroup differences: $Chi^2 = 30.40$, df = 1 (P < 0.00001), $I^2 = 96.7\%$

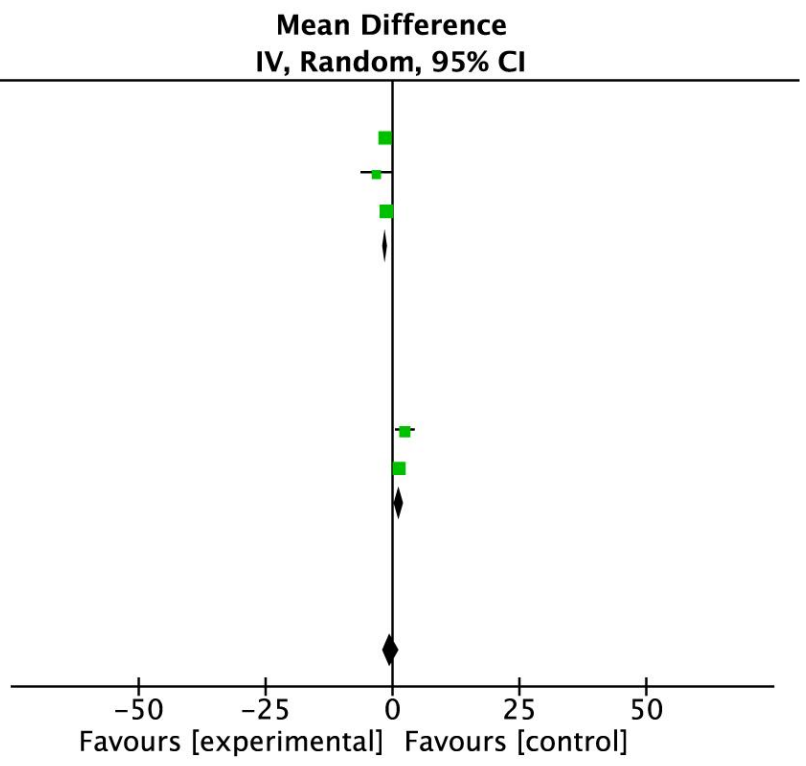

S16 Fig. Random-effects forest plot of short physical performance battery.

Study or Subgroup	Experimental Mean	Experimental SD	Experimental Total	Control Mean	Control SD	Control Total	Weight	Mean Difference IV, Random, 95% CI
Jingtao Zhou (2022)	9	1.41	13	7.23	2.98	13	9.7%	1.77 [−0.02, 3.56]
Na Peng et al.(2024)	9.12	2.24	63	7.85	2.13	63	53.4%	1.27 [0.51, 2.03]
Zhizhen Liu (2020)	10.7	1.23	18	9.3	1.56	18	36.9%	1.40 [0.48, 2.32]
**Total (95% CI)**			**94**			**94**	**100.0%**	**1.37 [0.81, 1.92]**

Heterogeneity: $Tau^2 = 0.00$; $Chi^2 = 0.26$, df = 2 (P = 0.88); $I^2 = 0\%$
Test for overall effect: Z = 4.80 (P < 0.00001)

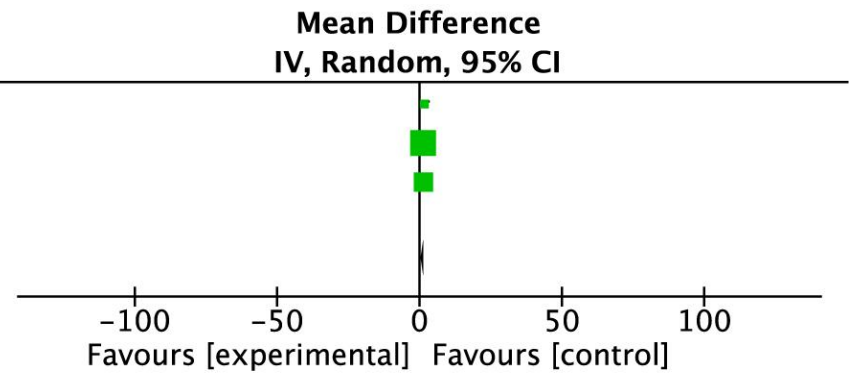

Supplement: Supplementary file 4 [file Supplementary_file_11.pdf]
