# Supplementary material for: Traditional Chinese exercises on muscle strength, muscle mass, and physical function in older adults with sarcopenia: a systematic review and meta-analysis
Source: Front Med (Lausanne). 2026 Aug 5;13:1841875. doi: 10.3389/fmed.2026.1841875 (PMC13487372; doi:10.3389/fmed.2026.1841875)

S5 Fig. Forest plot of handgip stength.

| Study or Subgroup | Experimental Mean | Experimental SD | Experimental Total | Control Mean | Control SD | Control Total | Weight | Mean Difference IV, Fixed, 95% CI |
|---|---|---|---|---|---|---|---|---|
| Barbara Morawin et al.(2021) | 22.1 | 4.3 | 40 | 21.5 | 3.9 | 40 | 1.1% | 0.60 [−1.20, 2.40] |
| Jingtao Zhou (2022) | 13.58 | 3.99 | 13 | 13.42 | 3.79 | 13 | 0.4% | 0.16 [−2.83, 3.15] |
| Jing Zhang (2025) | 15.04 | 5.07 | 18 | 14.31 | 2.29 | 18 | 0.6% | 0.73 [−1.84, 3.30] |
| Na Peng et al.(2024) | 27.1 | 14.03 | 63 | 23.01 | 4.11 | 63 | 0.3% | 4.09 [0.48, 7.70] |
| Shichun He (2024 ） | 21.44 | 2.83 | 23 | 19.22 | 3.29 | 23 | 1.2% | 2.22 [0.45, 3.99] |
| Song Zhu (2025) | 18.26 | 3 | 16 | 18.17 | 3.27 | 16 | 0.8% | 0.09 [−2.08, 2.26] |
| Supo Zhou et al.(2020) | 19.54 | 8.23 | 20 | 18.41 | 5.68 | 20 | 0.2% | 1.13 [−3.25, 5.51] |
| Tao Zhang et al.(2025) | 23.2 | 6 | 60 | 20.9 | 5.9 | 60 | 0.8% | 2.30 [0.17, 4.43] |
| Xijing Liu et al.(2024) | 20.46 | 1.15 | 60 | 19.11 | 0.21 | 60 | 42.3% | 1.35 [1.05, 1.65] |
| Xuechen Xu et al.(2022) | 20.74 | 0.45 | 30 | 19.78 | 0.61 | 30 | 50.3% | 0.96 [0.69, 1.23] |
| Yaqiong Zhu et al.(2019) | 15.29 | 5.38 | 24 | 15.16 | 4.52 | 27 | 0.5% | 0.13 [−2.62, 2.88] |
| Yongjun Zhao et al.(2016) | 23.77 | 6.88 | 6 | 21.92 | 6.65 | 6 | 0.1% | 1.85 [−5.81, 9.51] |
| Zhizhen Liu (2020) | 16.82 | 2.78 | 18 | 16.58 | 2.2 | 18 | 1.4% | 0.24 [−1.40, 1.88] |
| **Total (95% CI)** | | | **391** | | | **394** | **100.0%** | **1.13 [0.94, 1.32]** |

Heterogeneity: $Chi^2 = 12.21$, df = 12 (P = 0.43); $I^2 = 2\%$
Test for overall effect: Z = 11.52 (P < 0.00001)

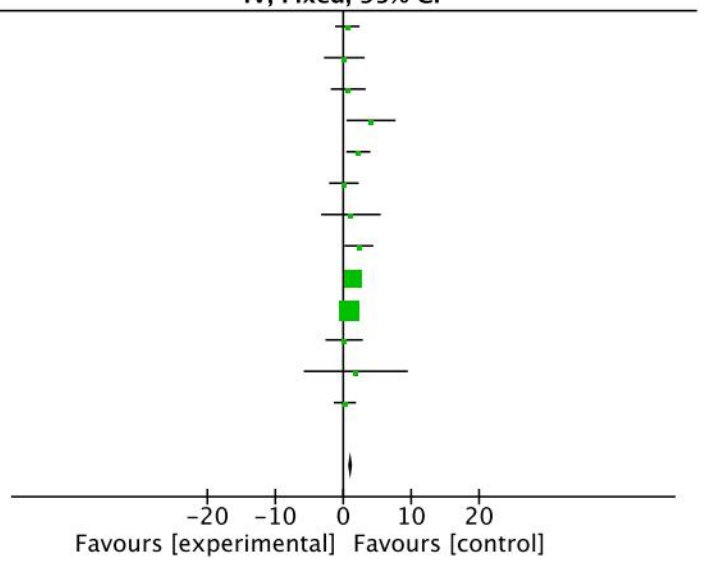


S5.1 Fig. Forest plot of handgrip strength for group subgroup analyses (exercise methods).

| Study or Subgroup | Experimental Mean | Experimental SD | Experimental Total | Control Mean | Control SD | Control Total | Weight | Mean Difference IV, Fixed, 95% CI |
|---|---|---|---|---|---|---|---|---|
| **1.1.1 Tai Chi** | | | | | | | | |
| Barbara Morawin et al.(2021) | 22.1 | 4.3 | 40 | 21.5 | 3.9 | 40 | 1.1% | 0.60 [−1.20, 2.40] |
| Shichun He (2024 ） | 21.44 | 2.83 | 23 | 19.22 | 3.29 | 23 | 1.2% | 2.22 [0.45, 3.99] |
| Yaqiong Zhu et al.(2019) | 15.29 | 5.38 | 24 | 15.16 | 4.52 | 27 | 0.5% | 0.13 [−2.62, 2.88] |
| Zhizhen Liu (2020) | 16.82 | 2.78 | 18 | 16.58 | 2.2 | 18 | 1.4% | 0.24 [−1.40, 1.88] |
| **Subtotal (95% CI)** | | | **105** | | | **108** | **4.2%** | **0.88 [−0.06, 1.82]** |
| Heterogeneity: $Chi^2 = 3.16$, df = 3 (P = 0.37); $I^2 = 5\%$ | | | | | | | | |
| Test for overall effect: Z = 1.84 (P = 0.07) | | | | | | | | |
| **1.1.2 Baduanjin** | | | | | | | | |
| Jingtao Zhou (2022) | 13.58 | 3.99 | 13 | 13.42 | 3.79 | 13 | 0.4% | 0.16 [−2.83, 3.15] |
| Jing Zhang (2025) | 15.04 | 5.07 | 18 | 14.31 | 2.29 | 18 | 0.6% | 0.73 [−1.84, 3.30] |
| Na Peng et al.(2024) | 27.1 | 14.03 | 63 | 23.01 | 4.11 | 63 | 0.3% | 4.09 [0.48, 7.70] |
| Song Zhu (2025) | 18.26 | 3 | 16 | 18.17 | 3.27 | 16 | 0.8% | 0.09 [−2.08, 2.26] |
| Supo Zhou et al.(2020) | 19.54 | 8.23 | 20 | 18.41 | 5.68 | 20 | 0.2% | 1.13 [−3.25, 5.51] |
| Xijing Liu et al.(2024) | 20.46 | 1.15 | 60 | 19.11 | 0.21 | 60 | 42.3% | 1.35 [1.05, 1.65] |
| Xuechen Xu et al.(2022) | 20.74 | 0.45 | 30 | 19.78 | 0.61 | 30 | 50.3% | 0.96 [0.69, 1.23] |
| **Subtotal (95% CI)** | | | **220** | | | **220** | **94.9%** | **1.13 [0.93, 1.33]** |
| Heterogeneity: $Chi^2 = 7.59$, df = 6 (P = 0.27); $I^2 = 21\%$ | | | | | | | | |
| Test for overall effect: Z = 11.23 (P < 0.00001) | | | | | | | | |
| **1.1.3 Yi Jin Jing** | | | | | | | | |
| Tao Zhang et al.(2025) | 23.2 | 6 | 60 | 20.9 | 5.9 | 60 | 0.8% | 2.30 [0.17, 4.43] |
| Yongjun Zhao et al.(2016) | 23.77 | 6.88 | 6 | 21.92 | 6.65 | 6 | 0.1% | 1.85 [−5.81, 9.51] |
| **Subtotal (95% CI)** | | | **66** | | | **66** | **0.9%** | **2.27 [0.22, 4.32]** |
| Heterogeneity: $Chi^2 = 0.01$, df = 1 (P = 0.91); $I^2 = 0\%$ | | | | | | | | |
| Test for overall effect: Z = 2.17 (P = 0.03) | | | | | | | | |
| **Total (95% CI)** | | | **391** | | | **394** | **100.0%** | **1.13 [0.94, 1.32]** |

Heterogeneity: $Chi^2 = 12.21$, df = 12 (P = 0.43); $I^2 = 2\%$
Test for overall effect: Z = 11.52 (P < 0.00001)
Test for subgroup differences: $Chi^2 = 1.45$, df = 2 (P = 0.48), $I^2 = 0\%$

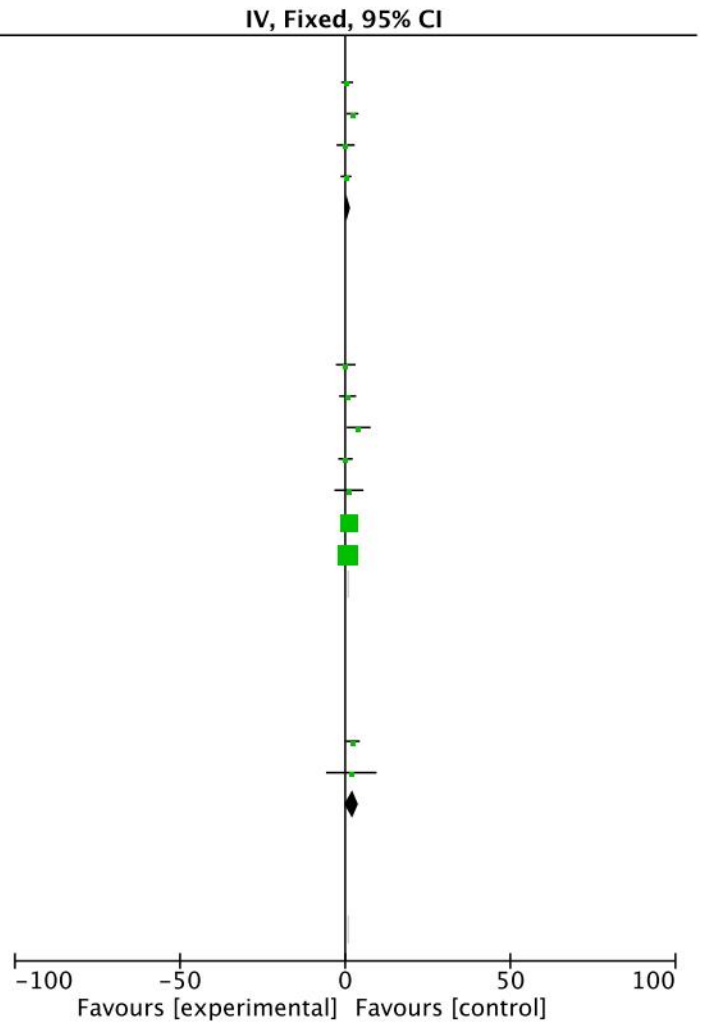


S5.2 Fig. Forest plot of handgrip strength for group subgroup analyses (length, weeks).

| Study or Subgroup | Experimental Mean | Experimental SD | Experimental Total | Control Mean | Control SD | Control Total | Weight | Mean Difference IV, Fixed, 95% CI |
|---|---|---|---|---|---|---|---|---|
| **1.2.1 ≤8** | | | | | | | | |
| Supo Zhou et al.(2020) | 19.54 | 8.23 | 20 | 18.41 | 5.68 | 20 | 0.2% | 1.13 [−3.25, 5.51] |
| Xijing Liu et al.(2024) | 20.46 | 1.15 | 60 | 19.11 | 0.21 | 60 | 42.3% | 1.35 [1.05, 1.65] |
| Yaqiong Zhu et al.(2019) | 15.29 | 5.38 | 24 | 15.16 | 4.52 | 27 | 0.5% | 0.13 [−2.62, 2.88] |
| Yongjun Zhao et al.(2016) | 23.77 | 6.88 | 6 | 21.92 | 6.65 | 6 | 0.1% | 1.85 [−5.81, 9.51] |
| **Subtotal (95% CI)** | | | **110** | | | **113** | **43.1%** | **1.34 [1.04, 1.63]** |
| Heterogeneity: $Chi^2 = 0.78$, df = 3 (P = 0.86); $I^2 = 0\%$ | | | | | | | | |
| Test for overall effect: Z = 8.93 (P < 0.00001) | | | | | | | | |
| **1.2.2 ＞8** | | | | | | | | |
| Barbara Morawin et al.(2021) | 22.1 | 4.3 | 40 | 21.5 | 3.9 | 40 | 1.1% | 0.60 [−1.20, 2.40] |
| Jingtao Zhou (2022) | 13.58 | 3.99 | 13 | 13.42 | 3.79 | 13 | 0.4% | 0.16 [−2.83, 3.15] |
| Jing Zhang (2025) | 15.04 | 5.07 | 18 | 14.31 | 2.29 | 18 | 0.6% | 0.73 [−1.84, 3.30] |
| Na Peng et al.(2024) | 27.1 | 14.03 | 63 | 23.01 | 4.11 | 63 | 0.3% | 4.09 [0.48, 7.70] |
| Shichun He (2024 ） | 21.44 | 2.83 | 23 | 19.22 | 3.29 | 23 | 1.2% | 2.22 [0.45, 3.99] |
| Song Zhu (2025) | 18.26 | 3 | 16 | 18.17 | 3.27 | 16 | 0.8% | 0.09 [−2.08, 2.26] |
| Tao Zhang et al.(2025) | 23.2 | 6 | 60 | 20.9 | 5.9 | 60 | 0.8% | 2.30 [0.17, 4.43] |
| Xuechen Xu et al.(2022) | 20.74 | 0.45 | 30 | 19.78 | 0.61 | 30 | 50.3% | 0.96 [0.69, 1.23] |
| Zhizhen Liu (2020) | 16.82 | 2.78 | 18 | 16.58 | 2.2 | 18 | 1.4% | 0.24 [−1.40, 1.88] |
| **Subtotal (95% CI)** | | | **281** | | | **281** | **56.9%** | **0.98 [0.72, 1.23]** |
| Heterogeneity: $Chi^2 = 8.15$, df = 8 (P = 0.42); $I^2 = 2\%$ | | | | | | | | |
| Test for overall effect: Z = 7.50 (P < 0.00001) | | | | | | | | |
| **Total (95% CI)** | | | **391** | | | **394** | **100.0%** | **1.13 [0.94, 1.32]** |

Heterogeneity: $Chi^2 = 12.21$, df = 12 (P = 0.43); $I^2 = 2\%$
Test for overall effect: Z = 11.52 (P < 0.00001)
Test for subgroup differences: $Chi^2 = 3.29$, df = 1 (P = 0.07), $I^2 = 69.6\%$

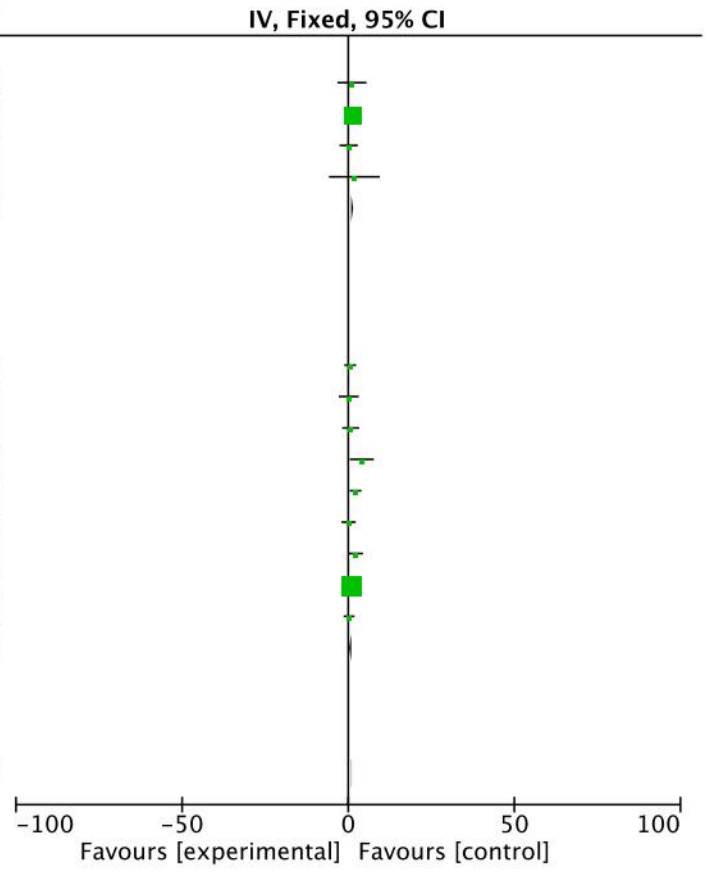

S5.3 Fig. Forest plot of handgrip strength for group subgroup analyses (frequency, sessionsweek).

| Study or Subgroup | Experimental Mean | Experimental SD | Experimental Total | Control Mean | Control SD | Control Total | Weight | Mean Difference IV, Fixed, 95% CI |
|---|---|---|---|---|---|---|---|---|
| **1.3.1 ≤3** | | | | | | | | |
| Barbara Morawin et al.(2021) | 22.1 | 4.3 | 40 | 21.5 | 3.9 | 40 | 1.1% | 0.60 [−1.20, 2.40] |
| Jingtao Zhou (2022) | 13.58 | 3.99 | 13 | 13.42 | 3.79 | 13 | 0.4% | 0.16 [−2.83, 3.15] |
| Jing Zhang (2025) | 15.04 | 5.07 | 18 | 14.31 | 2.29 | 18 | 0.6% | 0.73 [−1.84, 3.30] |
| Shichun He (2024 ) | 21.44 | 2.83 | 23 | 19.22 | 3.29 | 23 | 1.2% | 2.22 [0.45, 3.99] |
| Song Zhu (2025) | 18.26 | 3 | 16 | 18.17 | 3.27 | 16 | 0.8% | 0.09 [−2.08, 2.26] |
| Xijing Liu et al.(2024) | 20.46 | 1.15 | 60 | 19.11 | 0.21 | 60 | 42.3% | 1.35 [1.05, 1.65] |
| Xuechen Xu et al.(2022) | 20.74 | 0.45 | 30 | 19.78 | 0.61 | 30 | 50.3% | 0.96 [0.69, 1.23] |
| Yongjun Zhao et al.(2016) | 23.77 | 6.88 | 6 | 21.92 | 6.65 | 6 | 0.1% | 1.85 [−5.81, 9.51] |
| Zhizhen Liu (2020) | 16.82 | 2.78 | 18 | 16.58 | 2.2 | 18 | 1.4% | 0.24 [−1.40, 1.88] |
| **Subtotal (95% CI)** | | | **224** | | | **224** | **98.2%** | **1.12 [0.92, 1.31]** |
| Heterogeneity: $Chi^2 = 7.95$, df = 8 (P = 0.44); $I^2 = 0\%$ | | | | | | | | |
| Test for overall effect: Z = 11.28 (P < 0.00001) | | | | | | | | |
| **1.3.2 ＞3** | | | | | | | | |
| Na Peng et al.(2024) | 27.1 | 14.03 | 63 | 23.01 | 4.11 | 63 | 0.3% | 4.09 [0.48, 7.70] |
| Supo Zhou et al.(2020) | 19.54 | 8.23 | 20 | 18.41 | 5.68 | 20 | 0.2% | 1.13 [−3.25, 5.51] |
| Tao Zhang et al.(2025) | 23.2 | 6 | 60 | 20.9 | 5.9 | 60 | 0.8% | 2.30 [0.17, 4.43] |
| Yaqiong Zhu et al.(2019) | 15.29 | 5.38 | 24 | 15.16 | 4.52 | 27 | 0.5% | 0.13 [−2.62, 2.88] |
| **Subtotal (95% CI)** | | | **167** | | | **170** | **1.8%** | **1.86 [0.42, 3.30]** |
| Heterogeneity: $Chi^2 = 3.26$, df = 3 (P = 0.35); $I^2 = 8\%$ | | | | | | | | |
| Test for overall effect: Z = 2.53 (P = 0.01) | | | | | | | | |
| **Total (95% CI)** | | | **391** | | | **394** | **100.0%** | **1.13 [0.94, 1.32]** |
| Heterogeneity: $Chi^2 = 12.21$, df = 12 (P = 0.43); $I^2 = 2\%$ | | | | | | | | |
| Test for overall effect: Z = 11.52 (P < 0.00001) | | | | | | | | |
| Test for subgroup differences: $Chi^2 = 1.01$, df = 1 (P = 0.32), $I^2 = 0.6\%$ | | | | | | | | |

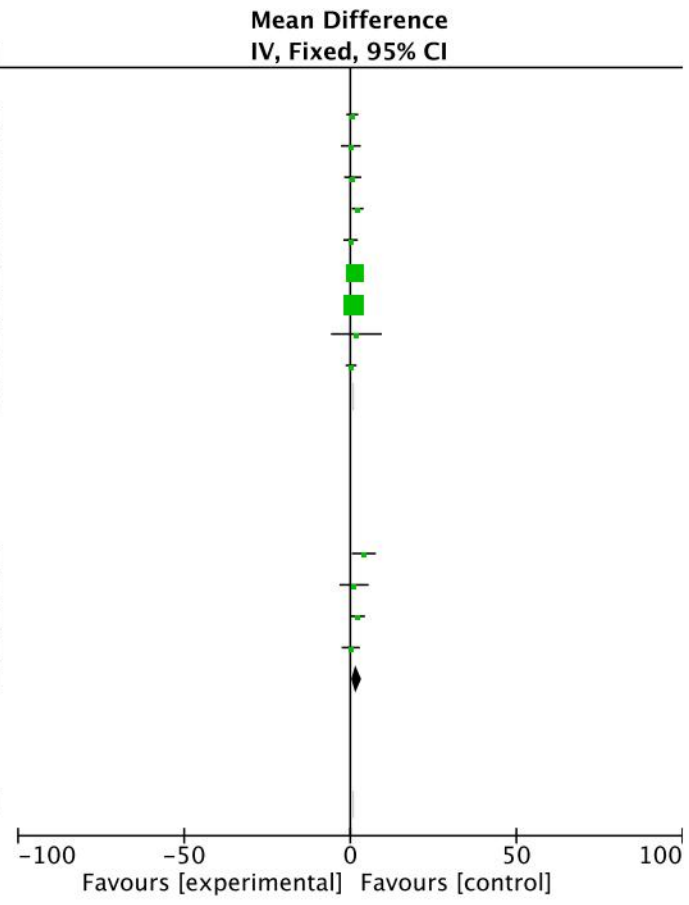


S5.4 Fig. Forest plot of handgrip strength for group subgroup analyses (session duration, minutes).

| Study or Subgroup | Experimental Mean | Experimental SD | Experimental Total | Control Mean | Control SD | Control Total | Weight | Mean Difference IV, Fixed, 95% CI |
|---|---|---|---|---|---|---|---|---|
| **1.4.1 ≤30** | | | | | | | | |
| Jing Zhang (2025) | 15.04 | 5.07 | 18 | 14.31 | 2.29 | 18 | 0.6% | 0.73 [−1.84, 3.30] |
| Na Peng et al.(2024) | 27.1 | 14.03 | 63 | 23.01 | 4.11 | 63 | 0.3% | 4.09 [0.48, 7.70] |
| Tao Zhang et al.(2025) | 23.2 | 6 | 60 | 20.9 | 5.9 | 60 | 0.8% | 2.30 [0.17, 4.43] |
| Xijing Liu et al.(2024) | 20.46 | 1.15 | 60 | 19.11 | 0.21 | 60 | 42.3% | 1.35 [1.05, 1.65] |
| Xuechen Xu et al.(2022) | 20.74 | 0.45 | 30 | 19.78 | 0.61 | 30 | 50.3% | 0.96 [0.69, 1.23] |
| **Subtotal (95% CI)** | | | **231** | | | **231** | **94.4%** | **1.15 [0.96, 1.35]** |
| Heterogeneity: $Chi^2 = 7.41$, df = 4 (P = 0.12); $I^2 = 46\%$ | | | | | | | | |
| Test for overall effect: Z = 11.42 (P < 0.00001) | | | | | | | | |
| **1.4.2 ＞30** | | | | | | | | |
| Barbara Morawin et al.(2021) | 22.1 | 4.3 | 40 | 21.5 | 3.9 | 40 | 1.1% | 0.60 [−1.20, 2.40] |
| Jingtao Zhou (2022) | 13.58 | 3.99 | 13 | 13.42 | 3.79 | 13 | 0.4% | 0.16 [−2.83, 3.15] |
| Shichun He (2024 ) | 21.44 | 2.83 | 23 | 19.22 | 3.29 | 23 | 1.2% | 2.22 [0.45, 3.99] |
| Song Zhu (2025) | 18.26 | 3 | 16 | 18.17 | 3.27 | 16 | 0.8% | 0.09 [−2.08, 2.26] |
| Supo Zhou et al.(2020) | 19.54 | 8.23 | 20 | 18.41 | 5.68 | 20 | 0.2% | 1.13 [−3.25, 5.51] |
| Yaqiong Zhu et al.(2019) | 15.29 | 5.38 | 24 | 15.16 | 4.52 | 27 | 0.5% | 0.13 [−2.62, 2.88] |
| Yongjun Zhao et al.(2016) | 23.77 | 6.88 | 6 | 21.92 | 6.65 | 6 | 0.1% | 1.85 [−5.81, 9.51] |
| Zhizhen Liu (2020) | 16.82 | 2.78 | 18 | 16.58 | 2.2 | 18 | 1.4% | 0.24 [−1.40, 1.88] |
| **Subtotal (95% CI)** | | | **160** | | | **163** | **5.6%** | **0.74 [−0.07, 1.55]** |
| Heterogeneity: $Chi^2 = 3.84$, df = 7 (P = 0.80); $I^2 = 0\%$ | | | | | | | | |
| Test for overall effect: Z = 1.79 (P = 0.07) | | | | | | | | |
| **Total (95% CI)** | | | **391** | | | **394** | **100.0%** | **1.13 [0.94, 1.32]** |
| Heterogeneity: $Chi^2 = 12.21$, df = 12 (P = 0.43); $I^2 = 2\%$ | | | | | | | | |
| Test for overall effect: Z = 11.52 (P < 0.00001) | | | | | | | | |
| Test for subgroup differences: $Chi^2 = 0.96$, df = 1 (P = 0.33), $I^2 = 0\%$ | | | | | | | | |

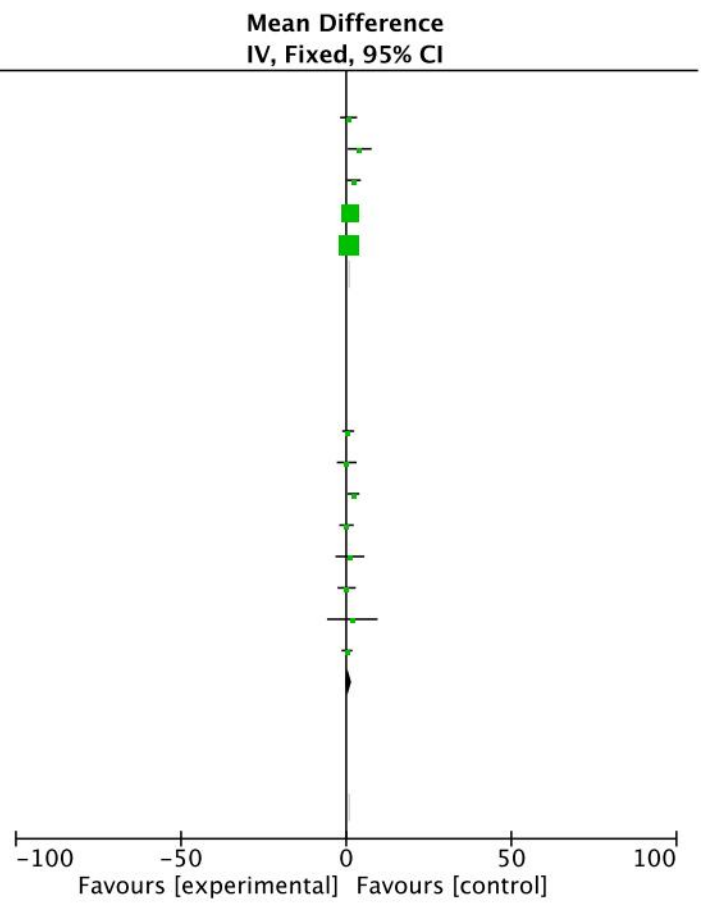

Supplement: Supplementary file 5 [file Supplementary_file_2.pdf]
