# Supplementary material for: Traditional Chinese exercises on muscle strength, muscle mass, and physical function in older adults with sarcopenia: a systematic review and meta-analysis
Source: Front Med (Lausanne). 2026 Aug 5;13:1841875. doi: 10.3389/fmed.2026.1841875 (PMC13487372; doi:10.3389/fmed.2026.1841875)

S6 Fig. Forest plot of 6-meter walk test.

Study or Subgroup	Experimental Mean	Experimental SD	Experimental Total	Control Mean	Control SD	Control Total	Weight	Mean Difference IV, Fixed, 95% CI
Barbara Morawin et al.(2021)	1.36	0.18	40	1.28	0.2	40	18.5%	0.08 [−0.00, 0.16]
Jingtao Zhou (2022)	0.87	0.07	13	0.73	0.1	13	29.3%	0.14 [0.07, 0.21]
Jing Zhang (2025)	0.58	0.19	18	0.46	0.29	18	5.0%	0.12 [−0.04, 0.28]
Shichun He (2024)	0.84	0.27	23	0.82	0.24	23	5.9%	0.02 [−0.13, 0.17]
Yongjun Zhao et al.(2016)	0.91	0.2	6	0.76	0.17	6	2.9%	0.15 [−0.06, 0.36]
Youdi Tao et al(2022)	0.96	0.12	37	0.82	0.31	36	11.0%	0.14 [0.03, 0.25]
Zhizhen Liu (2020)	1.02	0.1	18	0.88	0.11	18	27.3%	0.14 [0.07, 0.21]
**Total (95% CI)**			**155**			**154**	**100.0%**	**0.12 [0.09, 0.16]**

Heterogeneity: $Chi^2 = 3.53$, df = 6 (P = 0.74); $I^2 = 0\%$
Test for overall effect: Z = 6.61 (P < 0.00001)

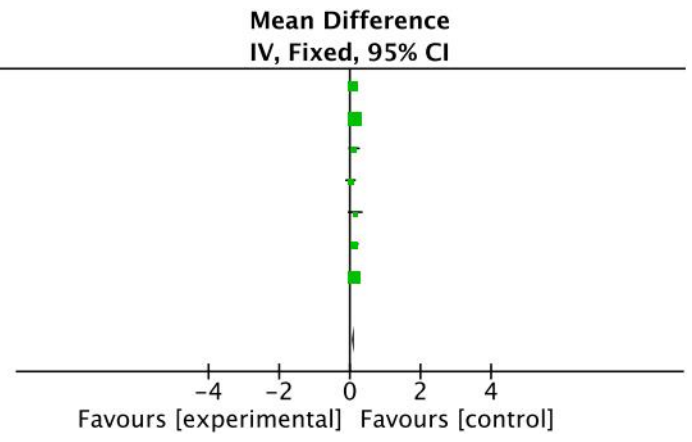

S6.1 Fig. Forest plot of 6-Meter walk test for group subgroup analyses (exercise methods).

Study or Subgroup	Experimental Mean	Experimental SD	Experimental Total	Control Mean	Control SD	Control Total	Weight	Mean Difference IV, Fixed, 95% CI
**1.1.1 Tai Chi**								
Barbara Morawin et al.(2021)	1.36	0.18	40	1.28	0.2	40	19.1%	0.08 [−0.00, 0.16]
Shichun He (2024)	0.84	0.27	23	0.82	0.24	23	6.1%	0.02 [−0.13, 0.17]
Youdi Tao et al(2022)	0.96	0.12	37	0.82	0.31	36	11.3%	0.14 [0.03, 0.25]
Zhizhen Liu (2020)	1.02	0.1	18	0.88	0.11	18	28.2%	0.14 [0.07, 0.21]
**Subtotal (95% CI)**			**118**			**117**	**64.7%**	**0.11 [0.07, 0.16]**
Heterogeneity: $Chi^2 = 2.95$, df = 3 (P = 0.40); $I^2 = 0\%$								
Test for overall effect: Z = 4.80 (P < 0.00001)								
**1.1.2 Baduanjin**								
Jingtao Zhou (2022)	0.87	0.07	13	0.73	0.1	13	30.2%	0.14 [0.07, 0.21]
Jing Zhang (2025)	0.58	0.19	18	0.46	0.29	18	5.2%	0.12 [−0.04, 0.28]
**Subtotal (95% CI)**			**31**			**31**	**35.3%**	**0.14 [0.08, 0.20]**
Heterogeneity: $Chi^2 = 0.05$, df = 1 (P = 0.82); $I^2 = 0\%$								
Test for overall effect: Z = 4.38 (P < 0.0001)								
**Total (95% CI)**			**149**			**148**	**100.0%**	**0.12 [0.08, 0.16]**

Heterogeneity: $Chi^2 = 3.45$, df = 5 (P = 0.63); $I^2 = 0\%$
Test for overall effect: Z = 6.46 (P < 0.00001)
Test for subgroup differences: $Chi^2 = 0.45$, df = 1 (P = 0.50), $I^2 = 0\%$

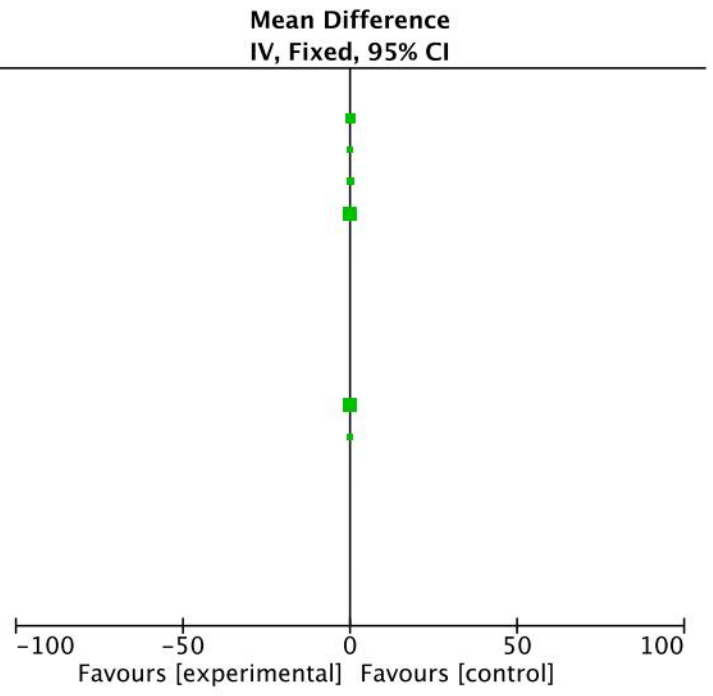

S6.2 Fig. Forest plot of 6-Meter walk test for group subgroup analyses (frequency, sessionsweek).

Study or Subgroup	Experimental Mean	Experimental SD	Experimental Total	Control Mean	Control SD	Control Total	Weight	Mean Difference IV, Fixed, 95% CI
**1.2.1 ≤ 8**								
Yongjun Zhao et al.(2016)	0.91	0.2	6	0.76	0.17	6	2.9%	0.15 [−0.06, 0.36]
**Subtotal (95% CI)**			**6**			**6**	**2.9%**	**0.15 [−0.06, 0.36]**
Heterogeneity: Not applicable								
Test for overall effect: Z = 1.40 (P = 0.16)								
**1.2.2 > 8**								
Barbara Morawin et al.(2021)	1.36	0.18	40	1.28	0.2	40	18.5%	0.08 [−0.00, 0.16]
Jingtao Zhou (2022)	0.87	0.07	13	0.73	0.1	13	29.3%	0.14 [0.07, 0.21]
Jing Zhang (2025)	0.58	0.19	18	0.46	0.29	18	5.0%	0.12 [−0.04, 0.28]
Shichun He (2024)	0.84	0.27	23	0.82	0.24	23	5.9%	0.02 [−0.13, 0.17]
Youdi Tao et al(2022)	0.96	0.12	37	0.82	0.31	36	11.0%	0.14 [0.03, 0.25]
Zhizhen Liu (2020)	1.02	0.1	18	0.88	0.11	18	27.3%	0.14 [0.07, 0.21]
**Subtotal (95% CI)**			**149**			**148**	**97.1%**	**0.12 [0.08, 0.16]**
Heterogeneity: $Chi^2 = 3.45$, df = 5 (P = 0.63); $I^2 = 0\%$								
Test for overall effect: Z = 6.46 (P < 0.00001)								
**Total (95% CI)**			**155**			**154**	**100.0%**	**0.12 [0.09, 0.16]**

Heterogeneity: $Chi^2 = 3.53$, df = 6 (P = 0.74); $I^2 = 0\%$
Test for overall effect: Z = 6.61 (P < 0.00001)
Test for subgroup differences: $Chi^2 = 0.08$, df = 1 (P = 0.78), $I^2 = 0\%$

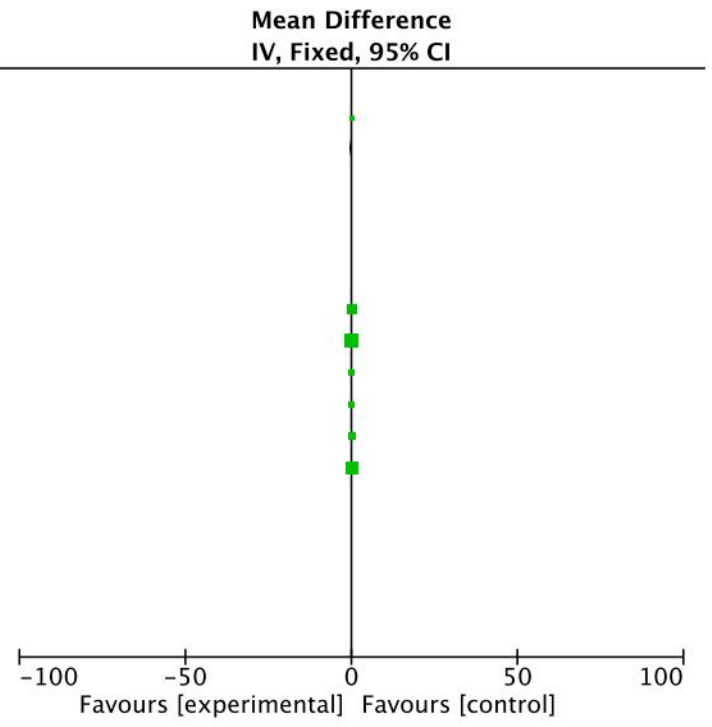

S6.3 Fig. Forest plot of 6-Meter walk test for group subgroup analyses (Session duration, minutes).

Study or Subgroup	Experimental Mean	Experimental SD	Experimental Total	Control Mean	Control SD	Control Total	Weight	Mean Difference IV, Fixed, 95% CI
**1.4.1 ≤ 40**								
Barbara Morawin et al.(2021)	1.36	0.18	40	1.28	0.2	40	18.5%	0.08 [−0.00, 0.16]
Jing Zhang (2025)	0.58	0.19	18	0.46	0.29	18	5.0%	0.12 [−0.04, 0.28]
Shichun He (2024)	0.84	0.27	23	0.82	0.24	23	5.9%	0.02 [−0.13, 0.17]
Yongjun Zhao et al.(2016)	0.91	0.2	6	0.76	0.17	6	2.9%	0.15 [−0.06, 0.36]
**Subtotal (95% CI)**			**87**			**87**	**32.4%**	**0.08 [0.02, 0.14]**
Heterogeneity: $Chi^2 = 1.30$, df = 3 (P = 0.73); $I^2 = 0\%$								
Test for overall effect: Z = 2.53 (P = 0.01)								
**1.4.2 > 40**								
Jingtao Zhou (2022)	0.87	0.07	13	0.73	0.1	13	29.3%	0.14 [0.07, 0.21]
Youdi Tao et al(2022)	0.96	0.12	37	0.82	0.31	36	11.0%	0.14 [0.03, 0.25]
Zhizhen Liu (2020)	1.02	0.1	18	0.88	0.11	18	27.3%	0.14 [0.07, 0.21]
**Subtotal (95% CI)**			**68**			**67**	**67.6%**	**0.14 [0.10, 0.18]**
Heterogeneity: $Chi^2 = 0.00$, df = 2 (P = 1.00); $I^2 = 0\%$								
Test for overall effect: Z = 6.28 (P < 0.00001)								
**Total (95% CI)**			**155**			**154**	**100.0%**	**0.12 [0.09, 0.16]**

Heterogeneity: $Chi^2 = 3.53$, df = 6 (P = 0.74); $I^2 = 0\%$
Test for overall effect: Z = 6.61 (P < 0.00001)
Test for subgroup differences: $Chi^2 = 2.23$, df = 1 (P = 0.14), $I^2 = 55.1\%$

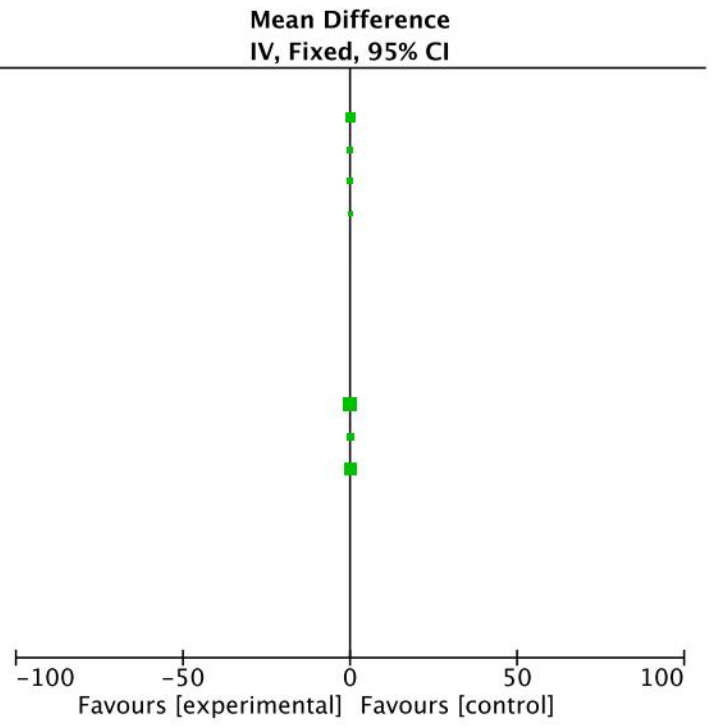

Supplement: Supplementary file 6 [file Supplementary_file_3.pdf]
