# Supplementary material for: Traditional Chinese exercises on muscle strength, muscle mass, and physical function in older adults with sarcopenia: a systematic review and meta-analysis
Source: Front Med (Lausanne). 2026 Aug 5;13:1841875. doi: 10.3389/fmed.2026.1841875 (PMC13487372; doi:10.3389/fmed.2026.1841875)

S9 Fig. Forest plot of sit-to-stand test.

| Study or Subgroup | Experimental Mean | Experimental SD | Experimental Total | Control Mean | Control SD | Control Total | Weight | Mean Difference IV, Fixed, 95% CI |
|---|---|---|---|---|---|---|---|---|
| **1.2.1 Five-Times-Sit-to-Stand Test** | | | | | | | | |
| Bingdun Huang et al. (2023) | 11.13 | 1.53 | 17 | 12.6 | 0.78 | 18 | 30.6% | −1.47 [−2.28, −0.66] |
| Jing Zhang (2025) | 15.59 | 4.05 | 18 | 18.79 | 5.44 | 18 | 2.1% | −3.20 [−6.33, −0.07] |
| Yaqiong Zhu et al.(2016) | 6.5 | 1.5 | 28 | 7.7 | 1.1 | 27 | 41.9% | −1.20 [−1.89, −0.51] |
| **Subtotal (95% CI)** | | | **63** | | | **63** | **74.6%** | **−1.37 [−1.89, −0.85]** |
| Heterogeneity: $Chi^2 = 1.60$, df = 2 (P = 0.45); $I^2 = 0\%$ | | | | | | | | |
| Test for overall effect: Z = 5.15 (P < 0.00001) | | | | | | | | |
| **1.2.2 30-Second Sit-to-Stand Test** | | | | | | | | |
| Supo Zhou et al.(2020) | 8.53 | 2.66 | 20 | 6.12 | 3.53 | 20 | 5.4% | 2.41 [0.47, 4.35] |
| Tao Zhang et al.(2025) | 13.6 | 2.7 | 60 | 12.3 | 2.9 | 60 | 20.1% | 1.30 [0.30, 2.30] |
| **Subtotal (95% CI)** | | | **80** | | | **80** | **25.4%** | **1.53 [0.64, 2.42]** |
| Heterogeneity: $Chi^2 = 0.99$, df = 1 (P = 0.32); $I^2 = 0\%$ | | | | | | | | |
| Test for overall effect: Z = 3.38 (P = 0.0007) | | | | | | | | |
| **Total (95% CI)** | | | **143** | | | **143** | **100.0%** | **−0.63 [−1.08, −0.18]** |
| Heterogeneity: $Chi^2 = 32.99$, df = 4 (P < 0.00001); $I^2 = 88\%$ | | | | | | | | |
| Test for overall effect: Z = 2.74 (P = 0.006) | | | | | | | | |
| Test for subgroup differences: $Chi^2 = 30.40$, df = 1 (P < 0.00001), $I^2 = 96.7\%$ | | | | | | | | |

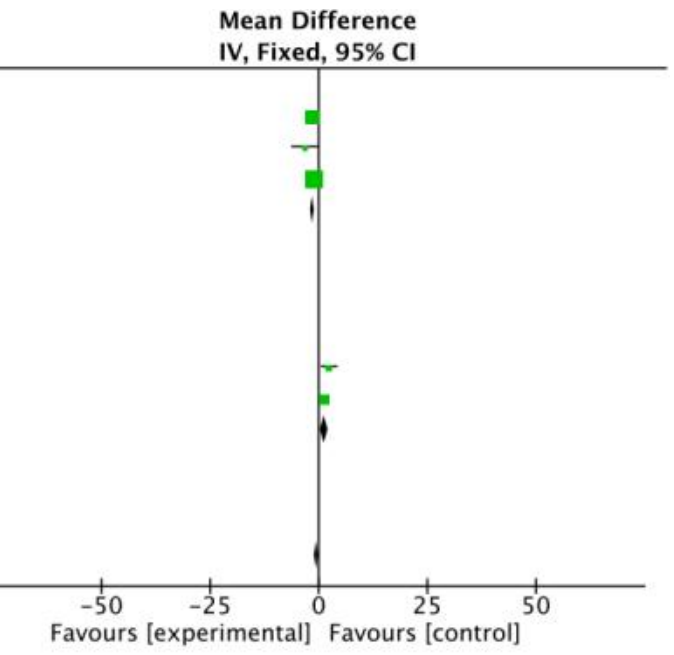


S10 Fig. Forest plot of short physical performance battery.

| Study or Subgroup | Experimental Mean | Experimental SD | Experimental Total | Control Mean | Control SD | Control Total | Weight | Mean Difference IV, Fixed, 95% CI |
|---|---|---|---|---|---|---|---|---|
| Jingtao Zhou (2022) | 9 | 1.41 | 13 | 7.23 | 2.98 | 13 | 9.7% | 1.77 [−0.02, 3.56] |
| Na Peng et al.(2024) | 9.12 | 2.24 | 63 | 7.85 | 2.13 | 63 | 53.4% | 1.27 [0.51, 2.03] |
| Zhizhen Liu (2020) | 10.7 | 1.23 | 18 | 9.3 | 1.56 | 18 | 36.9% | 1.40 [0.48, 2.32] |
| **Total (95% CI)** | | | **94** | | | **94** | **100.0%** | **1.37 [0.81, 1.92]** |
| Heterogeneity: $Chi^2 = 0.26$, df = 2 (P = 0.88); $I^2 = 0\%$ | | | | | | | | |
| Test for overall effect: Z = 4.80 (P < 0.00001) | | | | | | | | |

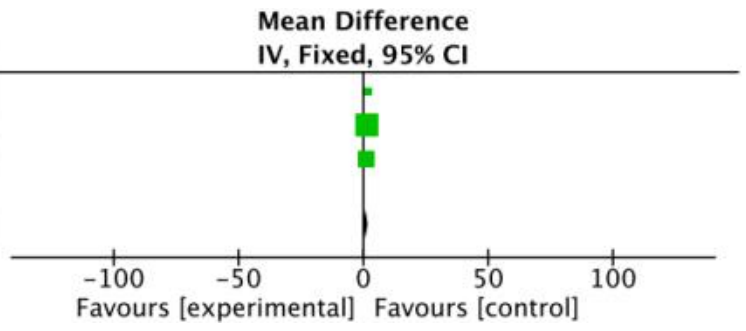

Supplement: Supplementary file 9 [file Supplementary_file_6.pdf]
