# Supplementary material for: Traditional Chinese exercises on muscle strength, muscle mass, and physical function in older adults with sarcopenia: a systematic review and meta-analysis
Source: Front Med (Lausanne). 2026 Aug 5;13:1841875. doi: 10.3389/fmed.2026.1841875 (PMC13487372; doi:10.3389/fmed.2026.1841875)

S11 Fig. Random-effects forest plot of grip strength.

Study or Subgroup	Experimental Mean	Experimental SD	Experimental Total	Control Mean	Control SD	Control Total	Weight	Mean Difference IV, Random, 95% CI
Barbara Morawin et al.(2021)	22.1	4.3	40	21.5	3.9	40	1.3%	0.60 [−1.20, 2.40]
Jingtao Zhou (2022)	13.58	3.99	13	13.42	3.79	13	0.5%	0.16 [−2.83, 3.15]
Jing Zhang (2025)	15.04	5.07	18	14.31	2.29	18	0.7%	0.73 [−1.84, 3.30]
Na Peng et al.(2024)	27.1	14.03	63	23.01	4.11	63	0.3%	4.09 [0.48, 7.70]
Shichun He (2024）	21.44	2.83	23	19.22	3.29	23	1.4%	2.22 [0.45, 3.99]
Song Zhu (2025)	18.26	3	16	18.17	3.27	16	0.9%	0.09 [−2.08, 2.26]
Supo Zhou et al.(2020)	19.54	8.23	20	18.41	5.68	20	0.2%	1.13 [−3.25, 5.51]
Tao Zhang et al.(2025)	23.2	6	60	20.9	5.9	60	0.9%	2.30 [0.17, 4.43]
Xijing Liu et al.(2024)	20.46	1.15	60	19.11	0.21	60	42.4%	1.35 [1.05, 1.65]
Xuechen Xu et al.(2022)	20.74	0.45	30	19.78	0.61	30	49.1%	0.96 [0.69, 1.23]
Yaqiong Zhu et al.(2019)	15.29	5.38	24	15.16	4.52	27	0.6%	0.13 [−2.62, 2.88]
Yongjun Zhao et al.(2016)	23.77	6.88	6	21.92	6.65	6	0.1%	1.85 [−5.81, 9.51]
Zhizhen Liu (2020)	16.82	2.78	18	16.58	2.2	18	1.6%	0.24 [−1.40, 1.88]
**Total (95% CI)**			**391**			**394**	**100.0%**	**1.13 [0.92, 1.34]**

Heterogeneity: $Tau^2 = 0.00$; $Chi^2 = 12.21$, df = 12 (P = 0.43); $I^2 = 2\%$
Test for overall effect: Z = 10.70 (P < 0.00001)

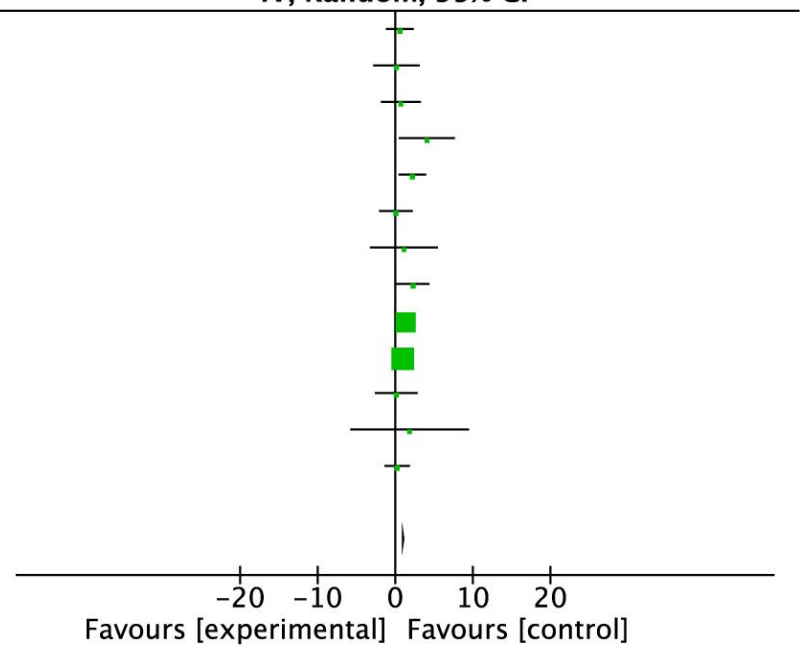

S11.1 Fig. Random-effects forest plot of handgrip strength for group subgroup analyses (exercise methods).

Study or Subgroup	Experimental Mean	Experimental SD	Experimental Total	Control Mean	Control SD	Control Total	Weight	Mean Difference IV, Random, 95% CI
**1.1.1 Tai Chi**								
Barbara Morawin et al.(2021)	22.1	4.3	40	21.5	3.9	40	1.3%	0.60 [−1.20, 2.40]
Shichun He (2024）	21.44	2.83	23	19.22	3.29	23	1.4%	2.22 [0.45, 3.99]
Yaqiong Zhu et al.(2019)	15.29	5.38	24	15.16	4.52	27	0.6%	0.13 [−2.62, 2.88]
Zhizhen Liu (2020)	16.82	2.78	18	16.58	2.2	18	1.6%	0.24 [−1.40, 1.88]
**Subtotal (95% CI)**			**105**			**108**	**4.9%**	**0.88 [−0.09, 1.85]**
Heterogeneity: $Tau^2 = 0.05$; $Chi^2 = 3.16$, df = 3 (P = 0.37); $I^2 = 5\%$								
Test for overall effect: Z = 1.78 (P = 0.07)								
**1.1.2 Baduanjin**								
Jingtao Zhou (2022)	13.58	3.99	13	13.42	3.79	13	0.5%	0.16 [−2.83, 3.15]
Jing Zhang (2025)	15.04	5.07	18	14.31	2.29	18	0.7%	0.73 [−1.84, 3.30]
Na Peng et al.(2024)	27.1	14.03	63	23.01	4.11	63	0.3%	4.09 [0.48, 7.70]
Song Zhu (2025)	18.26	3	16	18.17	3.27	16	0.9%	0.09 [−2.08, 2.26]
Supo Zhou et al.(2020)	19.54	8.23	20	18.41	5.68	20	0.2%	1.13 [−3.25, 5.51]
Xijing Liu et al.(2024)	20.46	1.15	60	19.11	0.21	60	42.4%	1.35 [1.05, 1.65]
Xuechen Xu et al.(2022)	20.74	0.45	30	19.78	0.61	30	49.1%	0.96 [0.69, 1.23]
**Subtotal (95% CI)**			**220**			**220**	**94.1%**	**1.13 [0.83, 1.44]**
Heterogeneity: $Tau^2 = 0.03$; $Chi^2 = 7.59$, df = 6 (P = 0.27); $I^2 = 21\%$								
Test for overall effect: Z = 7.23 (P < 0.00001)								
**1.1.3 Yi Jin Jing**								
Tao Zhang et al.(2025)	23.2	6	60	20.9	5.9	60	0.9%	2.30 [0.17, 4.43]
Yongjun Zhao et al.(2016)	23.77	6.88	6	21.92	6.65	6	0.1%	1.85 [−5.81, 9.51]
**Subtotal (95% CI)**			**66**			**66**	**1.0%**	**2.27 [0.22, 4.32]**
Heterogeneity: $Tau^2 = 0.00$; $Chi^2 = 0.01$, df = 1 (P = 0.91); $I^2 = 0\%$								
Test for overall effect: Z = 2.17 (P = 0.03)								
**Total (95% CI)**			**391**			**394**	**100.0%**	**1.13 [0.92, 1.34]**

Heterogeneity: $Tau^2 = 0.00$; $Chi^2 = 12.21$, df = 12 (P = 0.43); $I^2 = 2\%$
Test for overall effect: Z = 10.70 (P < 0.00001)
Test for subgroup differences: $Chi^2 = 1.44$, df = 2 (P = 0.49), $I^2 = 0\%$

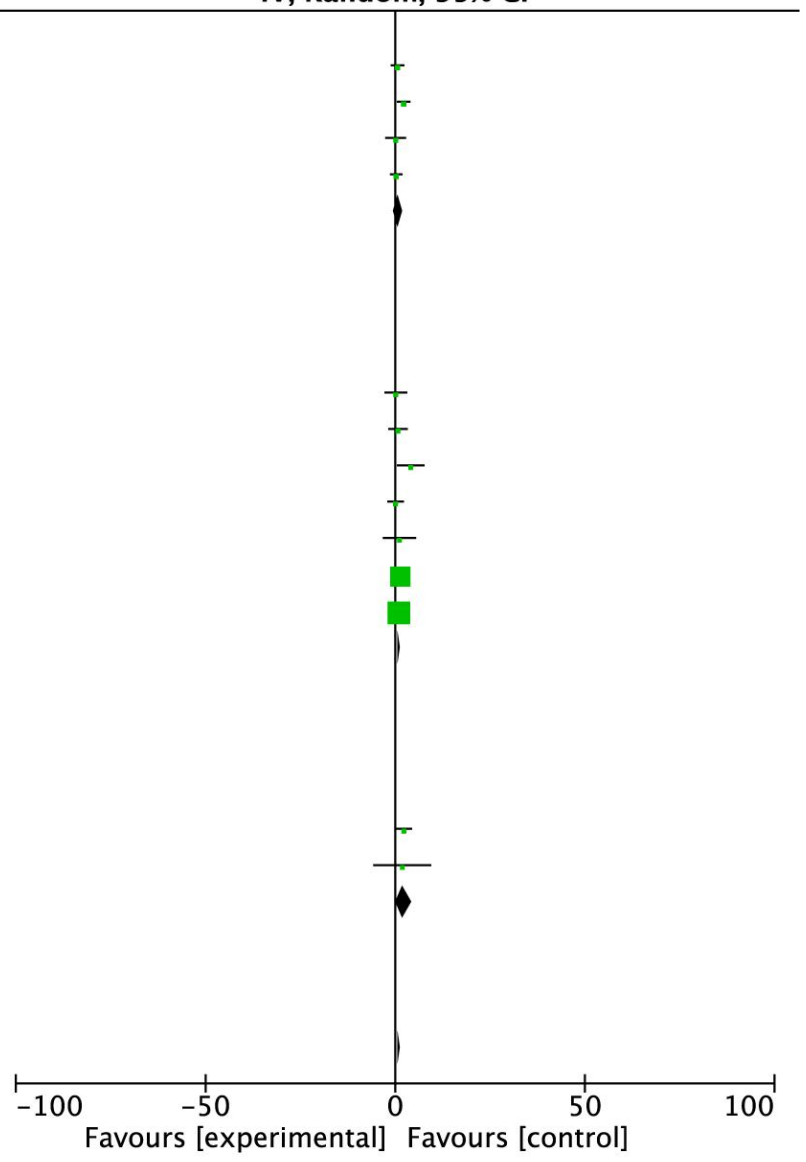

S11.2 Fig. Random-effects forest plot of handgrip strength for group subgroup analyses (length, weeks).

Study or Subgroup	Experimental Mean	Experimental SD	Experimental Total	Control Mean	Control SD	Control Total	Weight	Mean Difference IV, Random, 95% CI
**1.2.1 ≤8**								
Supo Zhou et al.(2020)	19.54	8.23	20	18.41	5.68	20	0.2%	1.13 [−3.25, 5.51]
Xijing Liu et al.(2024)	20.46	1.15	60	19.11	0.21	60	42.4%	1.35 [1.05, 1.65]
Yaqiong Zhu et al.(2019)	15.29	5.38	24	15.16	4.52	27	0.6%	0.13 [−2.62, 2.88]
Yongjun Zhao et al.(2016)	23.77	6.88	6	21.92	6.65	6	0.1%	1.85 [−5.81, 9.51]
**Subtotal (95% CI)**			**110**			**113**	**43.3%**	**1.34 [1.04, 1.63]**
Heterogeneity: $Tau^2 = 0.00$; $Chi^2 = 0.78$, df = 3 (P = 0.86); $I^2 = 0\%$								
Test for overall effect: Z = 8.93 (P < 0.00001)								
**1.2.2 ＞8**								
Barbara Morawin et al.(2021)	22.1	4.3	40	21.5	3.9	40	1.3%	0.60 [−1.20, 2.40]
Jingtao Zhou (2022)	13.58	3.99	13	13.42	3.79	13	0.5%	0.16 [−2.83, 3.15]
Jing Zhang (2025)	15.04	5.07	18	14.31	2.29	18	0.7%	0.73 [−1.84, 3.30]
Na Peng et al.(2024)	27.1	14.03	63	23.01	4.11	63	0.3%	4.09 [0.48, 7.70]
Shichun He (2024）	21.44	2.83	23	19.22	3.29	23	1.4%	2.22 [0.45, 3.99]
Song Zhu (2025)	18.26	3	16	18.17	3.27	16	0.9%	0.09 [−2.08, 2.26]
Tao Zhang et al.(2025)	23.2	6	60	20.9	5.9	60	0.9%	2.30 [0.17, 4.43]
Xuechen Xu et al.(2022)	20.74	0.45	30	19.78	0.61	30	49.1%	0.96 [0.69, 1.23]
Zhizhen Liu (2020)	16.82	2.78	18	16.58	2.2	18	1.6%	0.24 [−1.40, 1.88]
**Subtotal (95% CI)**			**281**			**281**	**56.7%**	**0.98 [0.67, 1.30]**
Heterogeneity: $Tau^2 = 0.01$; $Chi^2 = 8.15$, df = 8 (P = 0.42); $I^2 = 2\%$								
Test for overall effect: Z = 6.15 (P < 0.00001)								
**Total (95% CI)**			**391**			**394**	**100.0%**	**1.13 [0.92, 1.34]**

Heterogeneity: $Tau^2 = 0.00$; $Chi^2 = 12.21$, df = 12 (P = 0.43); $I^2 = 2\%$
Test for overall effect: Z = 10.70 (P < 0.00001)
Test for subgroup differences: $Chi^2 = 2.58$, df = 1 (P = 0.11), $I^2 = 61.2\%$

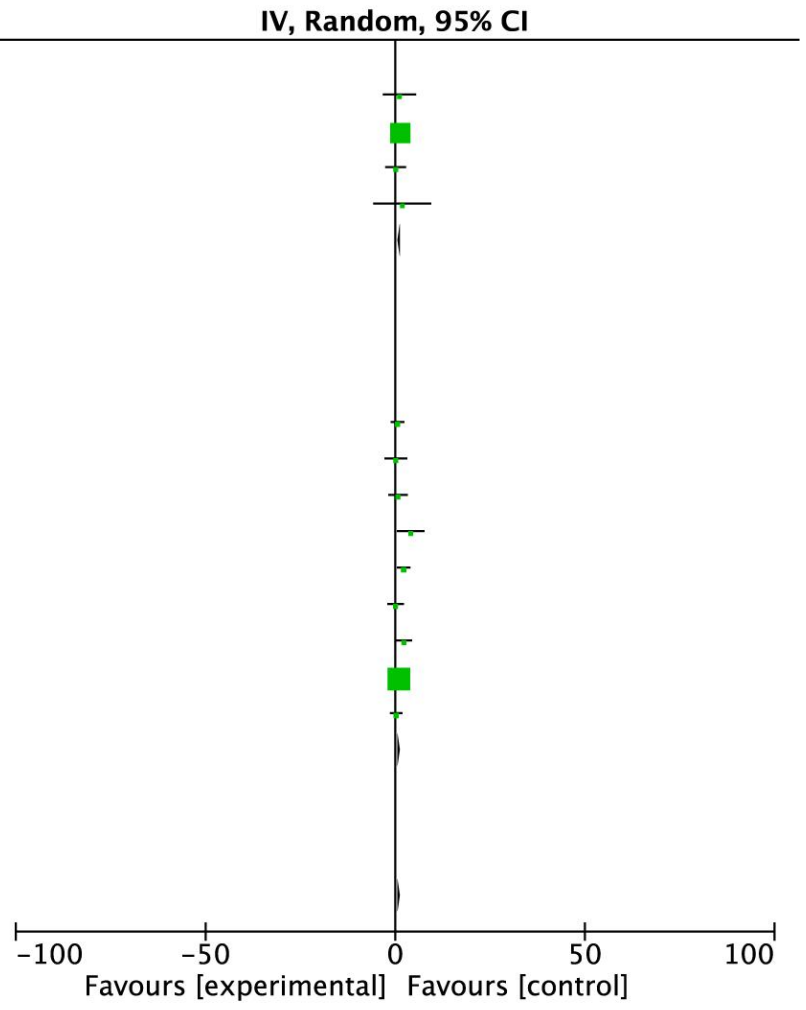

S11.3 Fig. Random-effects forest plot of handgrip strength for group subgroup analyses (frequency, sessions/week).

Study or Subgroup	Experimental Mean	Experimental SD	Experimental Total	Control Mean	Control SD	Control Total	Weight	Mean Difference IV, Random, 95% CI
**1.3.1 ≤3**								
Barbara Morawin et al.(2021)	22.1	4.3	40	21.5	3.9	40	1.3%	0.60 [−1.20, 2.40]
Jingtao Zhou (2022)	13.58	3.99	13	13.42	3.79	13	0.5%	0.16 [−2.83, 3.15]
Jing Zhang (2025)	15.04	5.07	18	14.31	2.29	18	0.7%	0.73 [−1.84, 3.30]
Shichun He (2024)	21.44	2.83	23	19.22	3.29	23	1.4%	2.22 [0.45, 3.99]
Song Zhu (2025)	18.26	3	16	18.17	3.27	16	0.9%	0.09 [−2.08, 2.26]
Xijing Liu et al.(2024)	20.46	1.15	60	19.11	0.21	60	42.4%	1.35 [1.05, 1.65]
Xuechen Xu et al.(2022)	20.74	0.45	30	19.78	0.61	30	49.1%	0.96 [0.69, 1.23]
Yongjun Zhao et al.(2016)	23.77	6.88	6	21.92	6.65	6	0.1%	1.85 [−5.81, 9.51]
Zhizhen Liu (2020)	16.82	2.78	18	16.58	2.2	18	1.6%	0.24 [−1.40, 1.88]
**Subtotal (95% CI)**			**224**			**224**	**97.9%**	**1.12 [0.92, 1.31]**
Heterogeneity: $Tau^2 = 0.00$; $Chi^2 = 7.95$, df = 8 (P = 0.44); $I^2 = 0\%$								
Test for overall effect: Z = 11.28 (P < 0.00001)								
**1.3.2 > 3**								
Na Peng et al.(2024)	27.1	14.03	63	23.01	4.11	63	0.3%	4.09 [0.48, 7.70]
Supo Zhou et al.(2020)	19.54	8.23	20	18.41	5.68	20	0.2%	1.13 [−3.25, 5.51]
Tao Zhang et al.(2025)	23.2	6	60	20.9	5.9	60	0.9%	2.30 [0.17, 4.43]
Yaqiong Zhu et al.(2019)	15.29	5.38	24	15.16	4.52	27	0.6%	0.13 [−2.62, 2.88]
**Subtotal (95% CI)**			**167**			**170**	**2.1%**	**1.86 [0.33, 3.39]**
Heterogeneity: $Tau^2 = 0.21$; $Chi^2 = 3.26$, df = 3 (P = 0.35); $I^2 = 8\%$								
Test for overall effect: Z = 2.39 (P = 0.02)								
**Total (95% CI)**			**391**			**394**	**100.0%**	**1.13 [0.92, 1.34]**
Heterogeneity: $Tau^2 = 0.00$; $Chi^2 = 12.21$, df = 12 (P = 0.43); $I^2 = 2\%$								
Test for overall effect: Z = 10.70 (P < 0.00001)								
Test for subgroup differences: $Chi^2 = 0.89$, df = 1 (P = 0.34), $I^2 = 0\%$								

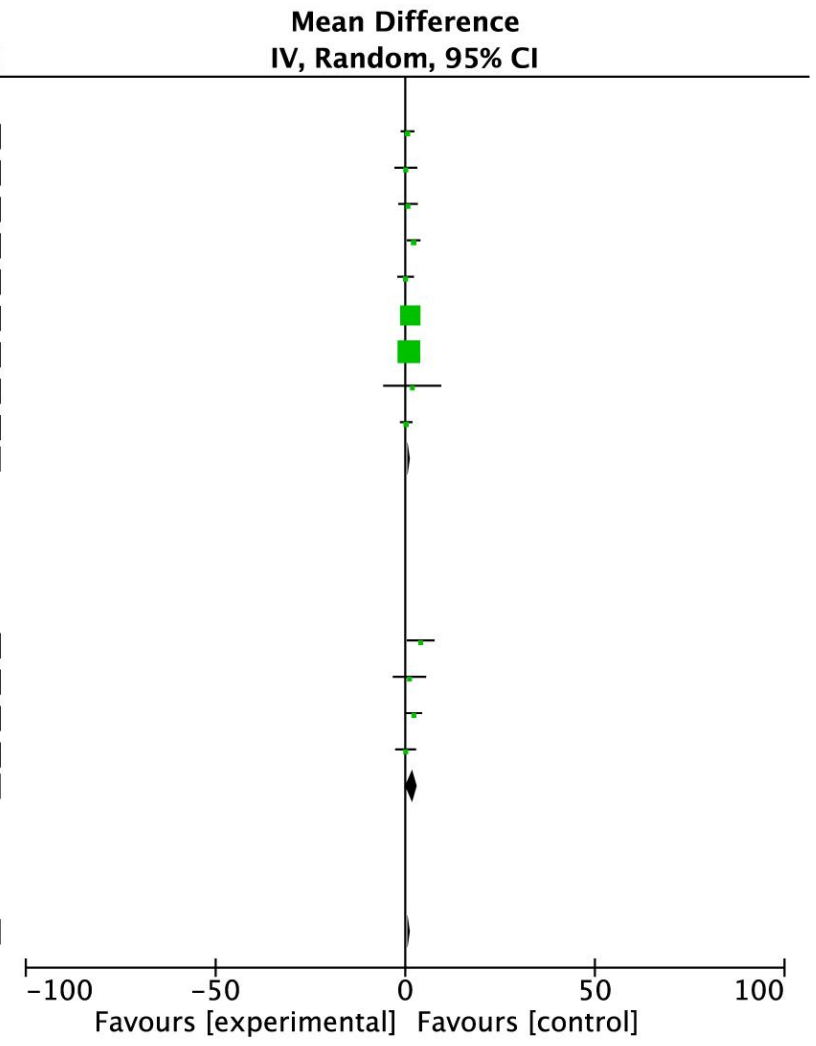

S11.4 Fig. Random-effects forest plot of handgrip strength for group subgroup analyses (session duration, minutes).

Study or Subgroup	Experimental Mean	Experimental SD	Experimental Total	Control Mean	Control SD	Control Total	Weight	Mean Difference IV, Random, 95% CI
**1.4.1 ≤30**								
Jing Zhang (2025)	15.04	5.07	18	14.31	2.29	18	0.7%	0.73 [−1.84, 3.30]
Na Peng et al.(2024)	27.1	14.03	63	23.01	4.11	63	0.3%	4.09 [0.48, 7.70]
Tao Zhang et al.(2025)	23.2	6	60	20.9	5.9	60	0.9%	2.30 [0.17, 4.43]
Xijing Liu et al.(2024)	20.46	1.15	60	19.11	0.21	60	42.4%	1.35 [1.05, 1.65]
Xuechen Xu et al.(2022)	20.74	0.45	30	19.78	0.61	30	49.1%	0.96 [0.69, 1.23]
**Subtotal (95% CI)**			**231**			**231**	**93.5%**	**1.21 [0.82, 1.61]**
Heterogeneity: $Tau^2 = 0.07$; $Chi^2 = 7.41$, df = 4 (P = 0.12); $I^2 = 46\%$								
Test for overall effect: Z = 5.97 (P < 0.00001)								
**1.4.2 > 30**								
Barbara Morawin et al.(2021)	22.1	4.3	40	21.5	3.9	40	1.3%	0.60 [−1.20, 2.40]
Jingtao Zhou (2022)	13.58	3.99	13	13.42	3.79	13	0.5%	0.16 [−2.83, 3.15]
Shichun He (2024)	21.44	2.83	23	19.22	3.29	23	1.4%	2.22 [0.45, 3.99]
Song Zhu (2025)	18.26	3	16	18.17	3.27	16	0.9%	0.09 [−2.08, 2.26]
Supo Zhou et al.(2020)	19.54	8.23	20	18.41	5.68	20	0.2%	1.13 [−3.25, 5.51]
Yaqiong Zhu et al.(2019)	15.29	5.38	24	15.16	4.52	27	0.6%	0.13 [−2.62, 2.88]
Yongjun Zhao et al.(2016)	23.77	6.88	6	21.92	6.65	6	0.1%	1.85 [−5.81, 9.51]
Zhizhen Liu (2020)	16.82	2.78	18	16.58	2.2	18	1.6%	0.24 [−1.40, 1.88]
**Subtotal (95% CI)**			**160**			**163**	**6.5%**	**0.74 [−0.07, 1.55]**
Heterogeneity: $Tau^2 = 0.00$; $Chi^2 = 3.84$, df = 7 (P = 0.80); $I^2 = 0\%$								
Test for overall effect: Z = 1.79 (P = 0.07)								
**Total (95% CI)**			**391**			**394**	**100.0%**	**1.13 [0.92, 1.34]**
Heterogeneity: $Tau^2 = 0.00$; $Chi^2 = 12.21$, df = 12 (P = 0.43); $I^2 = 2\%$								
Test for overall effect: Z = 10.70 (P < 0.00001)								
Test for subgroup differences: $Chi^2 = 1.07$, df = 1 (P = 0.30), $I^2 = 6.6\%$								

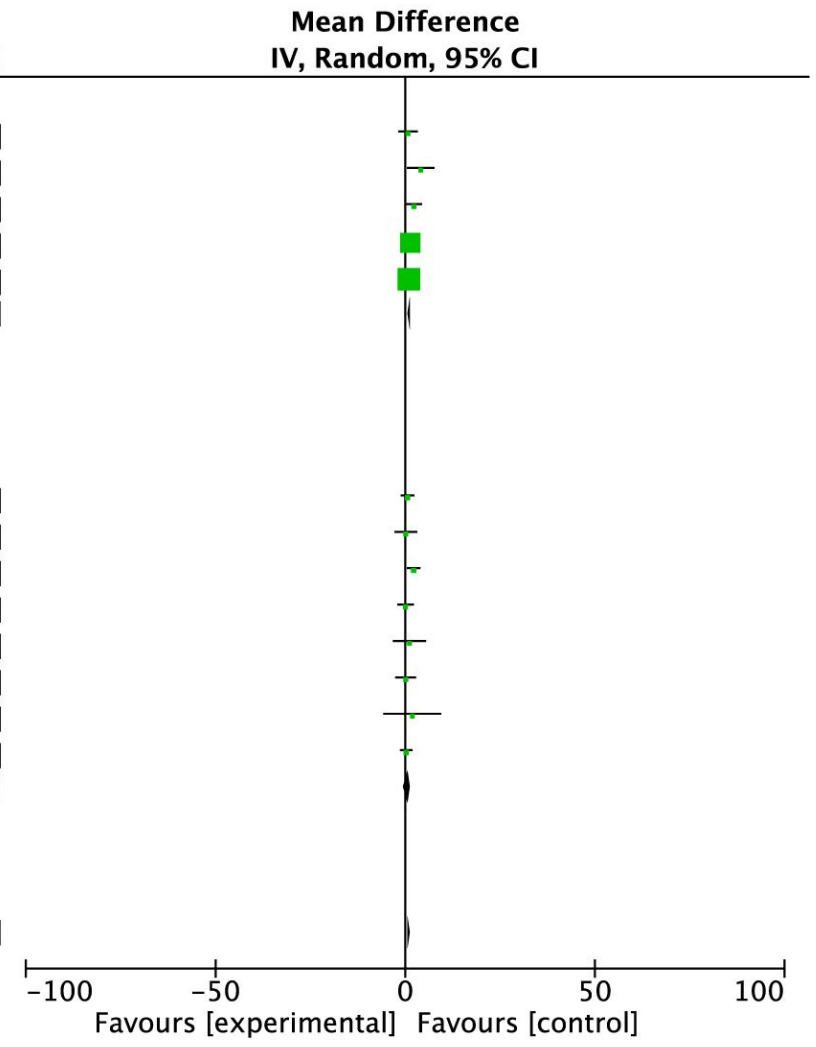

Supplement: Supplementary file 10 [file Supplementary_file_7.pdf]
