# Supplementary material for: Traditional Chinese exercises on muscle strength, muscle mass, and physical function in older adults with sarcopenia: a systematic review and meta-analysis
Source: Front Med (Lausanne). 2026 Aug 5;13:1841875. doi: 10.3389/fmed.2026.1841875 (PMC13487372; doi:10.3389/fmed.2026.1841875)

S13 Fig. Random-effects forest plot of appendicular skeletal muscle mass index.

Study or Subgroup	Experimental Mean	Experimental SD	Experimental Total	Control Mean	Control SD	Control Total	Weight	Mean Difference IV, Random, 95% CI
Barbara Morawin et al.(2021)	7	0.6	40	6.8	0.6	40	11.7%	0.20 [−0.06, 0.46]
Jingtao Zhou et al.(2022)	5.5	0.33	13	5.23	0.29	13	14.2%	0.27 [0.03, 0.51]
Jing Zhang et al.(2025)	5.44	0.35	18	5.404	0.37	18	14.6%	0.04 [−0.20, 0.27]
Shichun He et al.(2024)	5.14	0.68	23	4.71	0.81	23	4.3%	0.43 [−0.00, 0.86]
Song Zhu et al(2025)	6.51	0.33	16	6.4	0.37	16	13.7%	0.11 [−0.13, 0.35]
Tao Zhang et al.(2025)	6.06	0.97	60	5.76	0.91	60	7.1%	0.30 [−0.04, 0.64]
Yongjun Zhao et al.(2016)	6.25	0.93	6	6.37	0.49	6	1.1%	−0.12 [−0.96, 0.72]
Youdi Tao et al(2022)	6.81	0.35	37	6.57	0.33	36	33.2%	0.24 [0.08, 0.40]
**Total (95% CI)**			**213**			**212**	**100.0%**	**0.20 [0.11, 0.29]**

Heterogeneity: $Tau^2 = 0.00$; $Chi^2 = 4.96$, df = 7 (P = 0.66); $I^2 = 0\%$
Test for overall effect: Z = 4.37 (P < 0.0001)

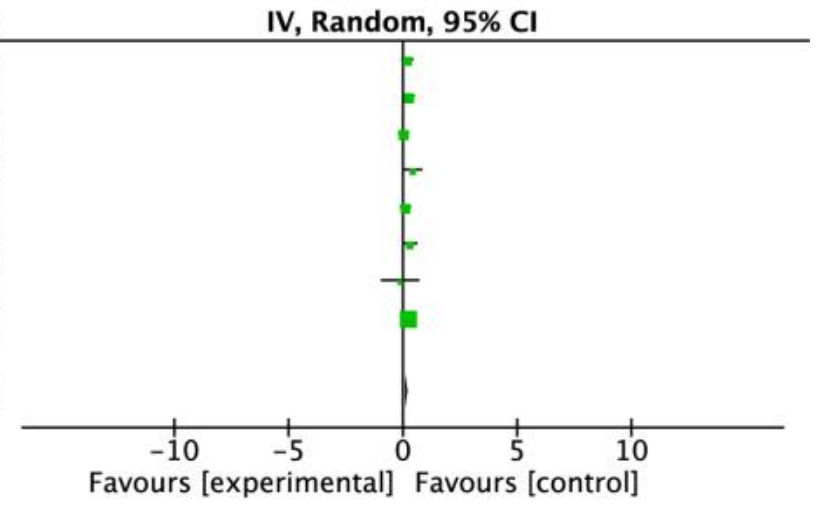

S13.1 Fig. Random-effects forest plot of appendicular skeletal muscle mass index for group subgroup analyses (exercise methods).

Study or Subgroup	Experimental Mean	Experimental SD	Experimental Total	Control Mean	Control SD	Control Total	Weight	Mean Difference IV, Random, 95% CI
**1.1.1 Tai Chi**								
Barbara Morawin et al.(2021)	7	0.6	40	6.8	0.6	40	11.7%	0.20 [−0.06, 0.46]
Shichun He et al.(2024)	5.14	0.68	23	4.71	0.81	23	4.3%	0.43 [−0.00, 0.86]
Youdi Tao et al(2022)	6.81	0.35	37	6.57	0.33	36	33.2%	0.24 [0.08, 0.40]
**Subtotal (95% CI)**			**100**			**99**	**49.2%**	**0.25 [0.12, 0.38]**
Heterogeneity: $Tau^2 = 0.00$; $Chi^2 = 0.82$, df = 2 (P = 0.66); $I^2 = 0\%$								
Test for overall effect: Z = 3.78 (P = 0.0002)								
**1.1.2 Baduanjin**								
Jingtao Zhou et al.(2022)	5.5	0.33	13	5.23	0.29	13	14.2%	0.27 [0.03, 0.51]
Jing Zhang et al.(2025)	5.44	0.35	18	5.404	0.37	18	14.6%	0.04 [−0.20, 0.27]
Song Zhu et al(2025)	6.51	0.33	16	6.4	0.37	16	13.7%	0.11 [−0.13, 0.35]
**Subtotal (95% CI)**			**47**			**47**	**42.5%**	**0.14 [−0.00, 0.28]**
Heterogeneity: $Tau^2 = 0.00$; $Chi^2 = 1.95$, df = 2 (P = 0.38); $I^2 = 0\%$								
Test for overall effect: Z = 1.96 (P = 0.05)								
**1.1.3 Yi Jin Jing**								
Tao Zhang et al.(2025)	6.06	0.97	60	5.76	0.91	60	7.1%	0.30 [−0.04, 0.64]
Yongjun Zhao et al.(2016)	6.25	0.93	6	6.37	0.49	6	1.1%	−0.12 [−0.96, 0.72]
**Subtotal (95% CI)**			**66**			**66**	**8.3%**	**0.24 [−0.07, 0.55]**
Heterogeneity: $Tau^2 = 0.00$; $Chi^2 = 0.83$, df = 1 (P = 0.36); $I^2 = 0\%$								
Test for overall effect: Z = 1.52 (P = 0.13)								
**Total (95% CI)**			**213**			**212**	**100.0%**	**0.20 [0.11, 0.29]**

Heterogeneity: $Tau^2 = 0.00$; $Chi^2 = 4.96$, df = 7 (P = 0.66); $I^2 = 0\%$
Test for overall effect: Z = 4.37 (P < 0.0001)
Test for subgroup differences: $Chi^2 = 1.37$, df = 2 (P = 0.50), $I^2 = 0\%$

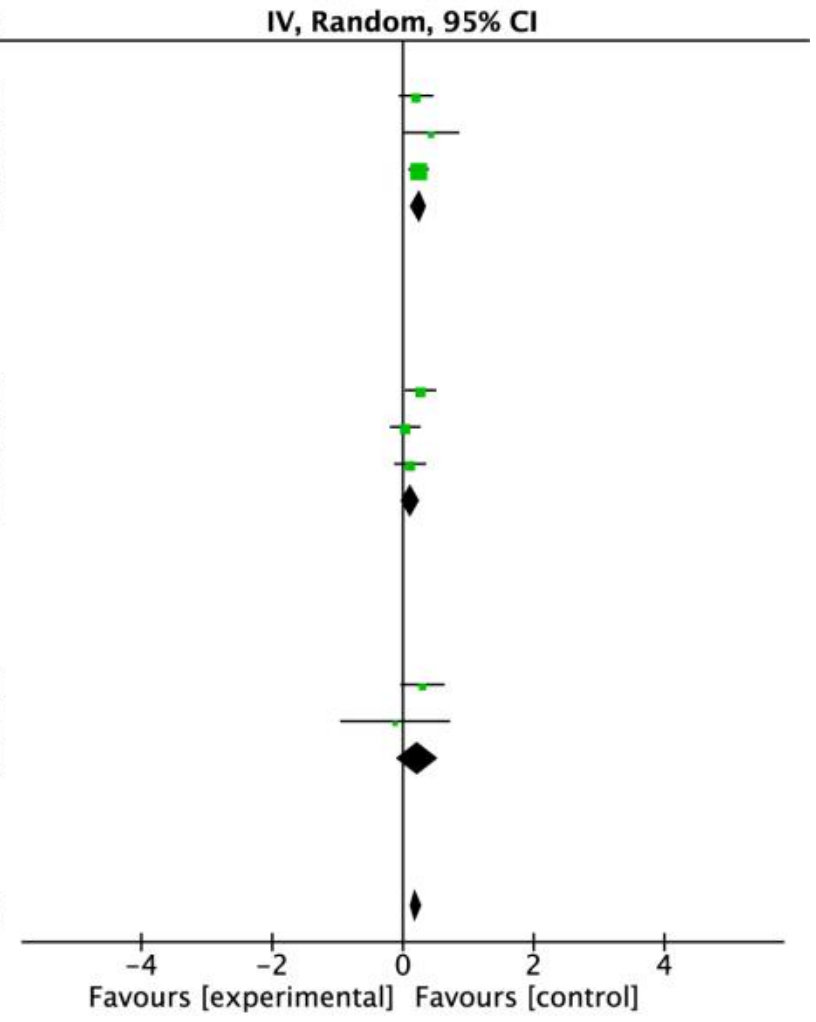

S13.2 Fig. Random-effects forest plot of appendicular skeletal muscle mass index for group subgroup analyses (Session duration, minutes).

Study or Subgroup	Experimental Mean	Experimental SD	Experimental Total	Control Mean	Control SD	Control Total	Weight	Mean Difference IV, Random, 95% CI
**1.3.1 ≤ 40**								
Barbara Morawin et al.(2021)	7	0.6	40	6.8	0.6	40	11.7%	0.20 [−0.06, 0.46]
Jing Zhang et al.(2025)	5.44	0.35	18	5.404	0.37	18	14.6%	0.04 [−0.20, 0.27]
Shichun He et al.(2024)	5.14	0.68	23	4.71	0.81	23	4.3%	0.43 [−0.00, 0.86]
Tao Zhang et al.(2025)	6.06	0.97	60	5.76	0.91	60	7.1%	0.30 [−0.04, 0.64]
Yongjun Zhao et al.(2016)	6.25	0.93	6	6.37	0.49	6	1.1%	−0.12 [−0.96, 0.72]
**Subtotal (95% CI)**			**147**			**147**	**38.9%**	**0.17 [0.03, 0.32]**
Heterogeneity: $Tau^2 = 0.00$; $Chi^2 = 3.71$, df = 4 (P = 0.45); $I^2 = 0\%$								
Test for overall effect: Z = 2.35 (P = 0.02)								
**1.3.2 > 40**								
Jingtao Zhou et al.(2022)	5.5	0.33	13	5.23	0.29	13	14.2%	0.27 [0.03, 0.51]
Song Zhu et al(2025)	6.51	0.33	16	6.4	0.37	16	13.7%	0.11 [−0.13, 0.35]
Youdi Tao et al(2022)	6.81	0.35	37	6.57	0.33	36	33.2%	0.24 [0.08, 0.40]
**Subtotal (95% CI)**			**66**			**65**	**61.1%**	**0.22 [0.10, 0.33]**
Heterogeneity: $Tau^2 = 0.00$; $Chi^2 = 1.02$, df = 2 (P = 0.60); $I^2 = 0\%$								
Test for overall effect: Z = 3.71 (P = 0.0002)								
**Total (95% CI)**			**213**			**212**	**100.0%**	**0.20 [0.11, 0.29]**

Heterogeneity: $Tau^2 = 0.00$; $Chi^2 = 4.96$, df = 7 (P = 0.66); $I^2 = 0\%$
Test for overall effect: Z = 4.37 (P < 0.0001)
Test for subgroup differences: $Chi^2 = 0.23$, df = 1 (P = 0.63), $I^2 = 0\%$

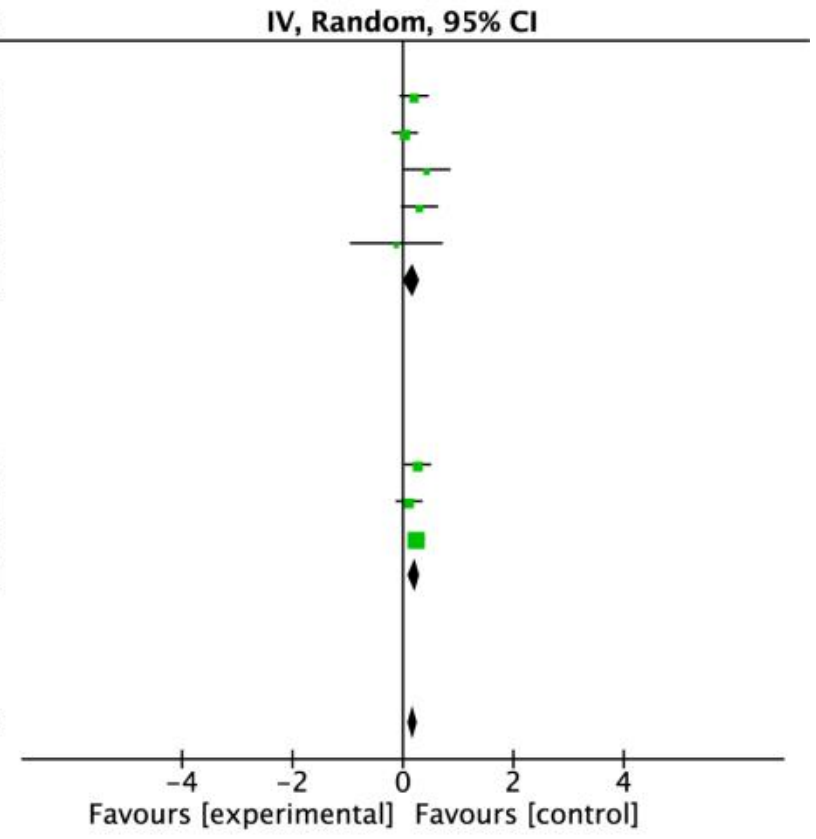

Supplement: Supplementary file 12 [file Supplementary_file_9.pdf]
