# Supplementary material for: Traditional Chinese exercises on muscle strength, muscle mass, and physical function in older adults with sarcopenia: a systematic review and meta-analysis
Source: Front Med (Lausanne). 2026 Aug 5;13:1841875. doi: 10.3389/fmed.2026.1841875 (PMC13487372; doi:10.3389/fmed.2026.1841875)
Supplement: Supplementary file 14 [file Data_Sheet_5.docx]

| **Outcome** | **No. RCTs** | **Risk of Bias** | **Inconsistency** | **Indirectness** | **Imprecision** | **Publication Bias** | **Overall Certainty** | **Comments** |
| --- | --- | --- | --- | --- | --- | --- | --- | --- |
| HS | 13 | Serious | Not serious | Serious | Not serious | Undetected | Moderate | Sample size is relatively large, effect is stable, I² = 2%. |
| 6m_GS | 7 | Serious | Not serious | Serious | Serious | Undetected | Low | Small number of studies; CI is relatively narrow but reliability is limited. |
| ASMI | 8 | Serious | Not serious | Serious | Serious | Undetected | Low | Effect size is small, sample is limited, generalizability is restricted. |
| TUGT | 5 | Serious | Not serious | Serious | Serious | Undetected | Low | Small sample; effect is clear but imprecise. |
| FTSST | 3 | Serious | Not serious | Serious | Serious | Undetected | Low | Very few studies; effect direction is clear but unstable. |
| 30s STS | 2 | Serious | Not serious | Serious | Serious | Undetected | Low | Extremely small sample; CI is relatively wide. |
| SPPB | 3 | Serious | Not serious | Serious | Serious | Undetected | Low | Small sample; effect direction is clear, CI is moderate. |

HS, handgrip strength; 6m_GS, 6-meter gait speed; ASMI, appendicular skeletal muscle mass index; TUGT, timed up-and-go test; FTSST, the five-times sit-to-stand test; 30s STS, the 30-second sit-to-stand test; SPPB, the short physical performance battery.
